# Rapid exometabolome footprinting combined with multivariate statistics: A powerful tool for bioprocess optimization

**DOI:** 10.1002/elsc.202300222

**Published:** 2024-03-05

**Authors:** Alexander Reiter, Lars Wesseling, Wolfgang Wiechert, Marco Oldiges

**Affiliations:** ^1^ Institute of Bio‐ and Geosciences IBG‐1: Biotechnology Forschungszentrum Jülich GmbH Jülich Germany; ^2^ Institute of Biotechnology RWTH Aachen University Aachen Germany; ^3^ Computational Systems Biotechnology RWTH Aachen University Aachen Germany

**Keywords:** bioprocess development, *Corynebacterium glutamicum*, dilute‐and‐shoot, flow‐injection‐analysis, l‐histidine, mass spectrometry

## Abstract

*Corynebacterium glutamicum* is used as an industrial platform organism for amino acid production. Previously, the organism was utilized to produce l‐histidine with research focusing on metabolic engineering approaches to increase titer and yield. Only a few studies have been published that provide information on bioprocess development, with media optimization and fed‐batch cultivation procedure being particularly promising areas. In this work, we show how experimental setups such as miniature cultivation technology, dynamic and time‐optimized LC‐MS/MS metabolic footprinting tools, and automated workflows for the detection of local and global metabolic patterns can significantly accelerate bioprocess development. Potential media bottlenecks in form of phosphate and magnesium availability were identified by sensitivity analysis in parallelized microscale cultivation assisted by lab automation. A rapid dilute‐and‐shoot flow‐injection‐analysis tandem mass spectrometry approach was used to cope with the resulting cultivation throughput and allowed to quantify amino acids with 1 min per sample. We were able to increase the l‐histidine titer of a *C. glutamicum* random mutagenesis mutant by a factor of 5.8 through process optimization while also identifying both known and previously unknown targets for additional strain improvements. The presented methodology can be seen as a supplement to traditional approaches in the field of bioprocess development.

**Abbreviations**
10‐fTHF: 10‐formyl‐tetrahydrofolate
5,10‐mTHF: 5,10‐methylene‐tetrahydrofolate
5‐mTHF: 5‐methyl‐tetrahydrofolate
AICAR: 1‐(5'‐phosphoribosyl)‐5‐amino‐4‐imidazolecarboxamide
Ala: l‐alanine
ATP: adenosine triphosphate
BS: backscatter
*C. glutamicum*
: *Corynebacterium glutamicum*
CDW: cell dry weight
Cys: l‐cysteine
DO: dissolved oxygen
DS‐FIA‐MS/MS: dilute‐and‐shoot flow‐injection‐analysis tandem mass spectrometry
FDR: false‐discovery‐rate, Benjamini‐Hochberg correction
Glc: d‐glucose
Glu: l‐glutamate
Gly: l‐glycine
HCA: hierarchical cluster analysis
His: l‐histidine
IDMS: isotope dilution mass spectrometry
LV: latent variable
MeOH: methanol
Met: l‐methionine
MOPS: 3‐(n‐morpholino)propanesulfonic acid
ORA: over representation analysis
PC: principal component
PCA: protocatechuic acid
PLS‐DA: partial least squares discriminant analysis
PTA: pathway topology analysis
QC: quality control
THF: tetrahydrofolate
Val: l‐valine
VIP: variable importance in projection

## 1 INTRODUCTION

*Corynebacterium glutamicum* (*C. glutamicum*) is a non‐sporulating gram‐positive soil bacterium belonging to the Actinobacteria phylum [1, 2]. It was found as a natural l‐glutamate (Glu) producer in the 1950s [3], and since was established as a platform organism in industrial biotechnology [4, 5, 6]. Because of its generally regarded as safe status, it is frequently used as a producer of food and feed additives such as amino acids [6, 7]. Although *C. glutamicum* is primarily used for large‐scale amino acid production [5, 8], extensive cellular functionality of the organism has made it accessible for bioprocess and metabolic engineering approaches for the production of organic acids [9, 10], proteins or enzymes [11, 12], biobased chemicals, intermediates, and healthcare products [13], biofuel components [14], hydroxybenzoic acids [15], isobutanol or polyhydroxybutyrate [16].

*C. glutamicum* has been extensively studied in terms of amino acid biosynthesis, with a particular emphasis on l‐lysine [17, 18, 19] and Glu [20]. It is, however, also utilized to produce other amino acids such as l‐valine (Val) [21], l‐leucine [22], l‐isoleucine [23], l‐cysteine (Cys) [24], Glu derivatives [25], and l‐histidine (His) [26]. His is one of the 20 proteinogenic amino acids and an essential amino acid for humans [27]. His is an interesting chemical for pharmaceutical uses due to its anti‐inflammatory properties [28, 29, 30, 31], positive effects on obesity [32, 33], and influence on blood pressure [34]. Furthermore, His is also utilized as a supplement in fish farming as an industrial food and feed ingredient [35].

Industrial production of His can be conducted by protein hydrolysis [36], synthesis [37], or biological transformation [38]. Because biological transformation facilitates the cost‐effective enantiopure generation of physiologically active compound [39], genetic modification of well‐known platform organisms was emphasized [40, 41]. Despite the fact that His biosynthesis is consistent across species [42, 43, 44, 45, 46], both *C. glutamicum* and *Escherichia coli* lack the histidine utilization system, which is found in many other bacteria to convert His to ammonia and Glu [47].

To enhance titer, yield, and productivity of His production, different metabolic engineering approaches such as random mutagenesis [26, 48], rational engineering [49, 50], and systems metabolic engineering [40] were used. His biosynthesis is a ten‐step metabolic pathway that is controlled by feedback inhibition [26, 51] and transcriptional attenuation [52]. The genetic alteration targets of this pathway include feedback regulation of the adenosine triphosphate (ATP) phosphoribosyltransferase *HisG* and suspected regulation of l‐histidinol dehydrogenase *HisD*, as well as the replacement of native promoters with stronger ones [40]. His biosynthesis is connected to purine biosynthesis directly through 1‐(5'‐phosphoribosyl)‐5‐amino‐4‐imidazolecarboxamide (AICAR) and indirectly through the cofactors tetrahydrofolate (THF) and 5,10‐methylene‐tetrahydrofolate (5,10‐mTHF). A further recycling reaction for 5,10‐mTHF from THF was provided by a heterologous transfer of the glycine cleavage system from *Corynebacterium jeikeium*, which eliminated l‐glycine (Gly) as a by‐product through cleavage of such into CO_2_ and ammonia [40]. Furthermore, an equimolar ATP concentration was reported to be required for efficient His production, which resulted in *purA* and *purB* overexpression in the purine pathway, allowing the conversion of resulting inosine monophosphate to adenosine monophosphate for ATP regeneration [40]. Because His biosynthesis is related directly to the pentose phosphate pathway via phosphoribosylpyrophosphate, carbon flux was adjusted by replacing the natural translational start codon of the *pgi* (glucose 6‐phosphate isomerase), to a weaker codon [40]. Specific growth rates for wild type *C. glutamicum* were reported to be around 0.4 h^−1^ [53, 54], however specific growth rates for His generating strains had been reported to be 0.22–0.34 h^−1^ [40, 50]. With respect to His production, titer of 3.5–17 mM [48, 50] and yields of 0.093 ± 0.003 mol_His_ mol_Glc_^−1^ [40] have been observed for a maximal theoretical yield of 0.51 mol_His_ mol_Glc_^−1^ at μ  =  0.1 h^−1^ [40].

Another technique for optimizing microbial cultivation procedures is bioprocess development, which involves determining appropriate process conditions such as pH, temperature, and medium composition [55]. The optimization of cultivation medium, in particular, is a time‐consuming and labor‐intensive operation [56]. Because most current research on *C. glutamicum* His production is focused on the establishment of production strains through specific genetic alterations, very few cultivation protocols had been reported. Previous cultivation procedures described were often carried out in shake flasks, glass tubes, and bioreactors with complex medium components such as molasses, vitamins, peptone, or yeast extracts [49, 57, 58]. Because such media compositions contain additional additives or substrate, the effects of basic mineral salt medium components on metabolism are overshadowed. Recent experiments used CGXII mineral salt medium in shaking flasks [40], microtiter flower plates [59], and microfluidic chips [48]. However, no systematic evaluation of mineral salt medium composition on His production with *C. glutamicum* is currently available. More research is needed, particularly on fed‐batch processes based on mineral salt medium.

In this study, we evaluated His production with *C. glutamicum* with CGXII media in a fed‐batch process. Based on sensitivity analysis for media components, limiting minimal media components on growth and production were identified in microtiter scale cultivations. Cultivation, sampling, and sample processing for subsequent analysis was conducted using an automated cultivation platform with minimal human interaction. Based on the identified bottlenecks, the fed‐batch procedure was optimized by media optimization with respect to His yield and titer. Dilute‐and‐shoot flow‐injection‐analysis tandem mass spectrometry (DS‐FIA‐MS/MS) [59] allowed for quantification of amino acids in form of main‐ and byproducts with an analysis time of 1 min per sample, being able to handle the throughput of microscale cultivation and bioreactor time‐resolved sampling. Advancement of the method [60] was used in a semi‐targeted metabolic footprinting approach to identify additional byproducts and their latent patterns based on a *C. glutamicum* genome model. This allowed to speculate about pathways related to future metabolic engineering targets based on the metabolic footprint. Overall, the workflow presented here comprises time‐efficient screening of limiting media components as pre‐work for time and cost‐intensive bioreactor fed‐batch cultivations.

## 2 MATERIALS AND METHODS

### 2.1 Chemicals

Unlabeled amino acids l‐aspartic acid, Glu, l‐serin, l‐asparagine, l‐threonine, l‐glutamin, l‐tyrosine, Gly, l‐proline, l‐alanine (Ala), l‐methionine (Met), Val, l‐phenylalanine, l‐isoleucine, l‐leucine, l‐tryptophane, His, l‐lysine, and l‐arginine were purchased from Sigma Aldrich (Schnelldorf, Germany). The cell free extract of ^13^C^15^N labeled amino acids was purchased from Sigma Aldrich (Schnelldorf, Germany). UPLC/MS‐grade methanol (MeOH) was obtained from Biosolve BV (Valkenswaard, Netherlands). LC‐MS grade ammonium acetate was purchased from Merck (Darmstadt, Germany). Acetic acid (Ph. Eur.) was obtained from Roth (Karlsruhe, Germany). Cultivation media components were purchased from Sigma Aldrich (Schnelldorf, Germany) or from Roth (Karlsruhe, Germany). LC‐MS grade water was obtained from a Milli‐Q water purification system (Merck Millipore, Burlington, MA, USA).

**PRACTICAL APPLICATION**

In this study, we demonstrate the power of high‐throughput cultivation technology, rapid metabolic screening tools, and advanced statical methods for current and future bioprocess development. To highlight the benefit and need of advancements in microbial screening technology, we selected a l‐histidine producing *C. glutamicum* strain obtained by random mutagenesis as an exemplary biological system to study. Micro‐scale cultivation technology embedded in laboratory robotics allowed to conduct a sensitivity analysis to identify media limitations and drastically reduce the amount of time for subsequent cost intensive lab‐scale bioreactor fed‐batch cultivations. Further metabolic footprinting experiments with time‐optimized multicomponent analytics, automatically developed for the specific biological system, provided insights about metabolic patterns, local pathway limitations, and optimization potential. The combination of parallelization, automation, and dynamic metabolic pattern screening provides the means for cost and time effective process development, which is valuable for research and development in academia and industry.

### 2.2 Strains, media, and feed

SenseUp GmbH (Juelich, Germany) provided a master cell bank of a *C. glutamicum* His producer strain obtained from random mutagenesis which was stored at −80°C. All cultivations were performed with defined CGXII media (CGXII^Ref^) based on [53] containing per L of distilled water: 20 g d‐glucose (Glc), 20 g (NH_4_)_2_SO_4_, 5 g urea, 1 g KH_2_PO_4_, 1 g K_2_HPO_4_, 13.25 mg CaCl_2_∙2 H_2_O, 0.25 g MgSO_4_∙7 H_2_O, 0.2 mg biotin, 30 mg protocatechuic acid (PCA), 10 mg FeSO_4_∙7 H_2_O, 10 mg MnSO_4_∙H_2_O, 1 mg ZnSO_4_∙7 H_2_O, 0.313 mg CuSO_4_∙5 H_2_O and 0.02 mg NiCl_2_∙6 H_2_O. For shaken cultures, the medium (CGXII^N^) was buffered with 42 g L^−1^ 3‐(n‐morpholino)propanesulfonic acid (MOPS). pH was adjusted to 7.0 with 4 M NaOH. Media for optimized cultivations was enriched 2‐fold with MgSO_4_∙7 H_2_O and 5‐fold with KH_2_PO_4_/K_2_HPO_4_ (CGXII^Mg,P^). Base feed solution contained 440 g L^−1^ Glc (Feed^Ref^) which was enriched with either 10 g L^−1^ MgSO_4_·7 H_2_O (Feed^Mg1^), 7 g L^−1^ MgSO_4_·7 H_2_O (Feed^Mg2^) or 7 g L^−1^ MgSO_4_·7 H_2_O and 105 g L^−1^ (NH_4_)_2_SO_4_ (Feed^Mg2,AMS^).

### 2.3 Cultivations

A working cell bank consisting of cryoconserved *C. glutamicum* His producer from random mutagenesis was used for all cultivations. Cryocultures were prepared from shake flask cultivations in CGXII^N^ inoculated from the master cell bank. Single‐stage shake flask cultivations were conducted in 100 mL baffled shake flasks with a cultivation volume of 10 mL, 250 rpm, shaking diameter of 25 mm, and 30°C for approx. 16 h. The cultivation was inoculated with an optical density (OD_600_) of approx. 0.1. Double‐stage shake flask cultivations were conducted in 500 mL baffled shake flasks with a cultivation volume of 50 mL and identical cultivation conditions. The culture was harvested and diluted 1:2 with 500 g L^−1^ sterile glycerol solution. Single‐use aliquots of 1 mL were frozen in sterile reactions tubes and stored at −80°C until use.

Microscale batch cultivations of the His producing *C. glutamicum* strain were performed at 30°C, 1300 rpm, 800 µL cultivation volume and 80% humidity in a BioLector system (m2p‐labs GmbH, Baesweiler, Germany) and 48 well flower plates with optodes for pH and dissolved oxygen (DO) measurement (MTP‐48‐BOH 1, m2p‐labs GmbH, Baesweiler, Germany). A single‐stage pre‐culture was used for inoculation of main cultures in CGXII^N^ with an optical density of approx. 0.1. The flower plate was manually sealed with sealing foil for automation (F‐GPRS48‐10, m2p‐labs GmbH, Baesweiler, Germany). The cultivation system is integrated into a liquid handling robot (Tecan Group, Maennedorf, Switzerland), which was used for automated sampling and sample processing. The end of cultivation or complete consumption of batch Glc was indicated by a sharp increase in DO signal. For sampling, the individual cultivations were harvested, deposited in a 2 mL deepwell plate and centrifuged for 5 min with 3220 g at 4°C. The supernatants were transferred to microtiter plates on a cooled carrier set to 4°C and manually sealed with self‐adhesive aluminum foil after all cultivations were finished, before being stored at −20°C.

Bioreactor cultivations were carried out in 1.8 L lab‐scale stirred tank reactors (DASGIP, Jülich, Germany), equipped with two Rushton Turbines, pH‐electrodes (Mettler Toledo, Gießen, Germany) and DO‐electrodes (Hamilton, Martinsried, Germany). Process control was conducted with DASGIP Control 4.0 (DASGIP, Jülich, Germany). DO was controlled at 30% saturation by constant gassing of 1 vvm air while stirrer speed was controlled from 400 to 1500 rpm. For the fed‐batch phase, gassing of air was dynamically controlled up to 2.5 vvm. pH was controlled at pH 7.0 using 25% (w w^−1^) ammonia solution and 7 M H_3_PO_4_. Cultivation temperature was controlled at 30°C and the initial reaction volume was 0.7 L. Bioreactor cultivations with the corresponding cultivation medium were inoculated with an OD_600_ of approx. 0.1 from double stage pre‐cultures in CGXII^N^. The fed‐batch phase was initiated after end of cultivation or complete consumption of batch Glc, which was indicated by a sharp increase in DO signal. For the fed‐batch phase, a constant feeding rate of 12.5 mL was applied.

### 2.4 Cell dry weight

Cell dry weight (CDW) was determined gravimetrically in 2 mL reaction tubes, which were pre‐weighted after active drying for 48 h at 80°C and passive cooling to room temperature in a desiccator. Cultivation samples of 1 mL were centrifuged for 10 min at 21.000 g. The cell‐free supernatants were distributed to microtiter plates, sealed with self‐adhesive aluminum foil, and stored at −20°C for further analysis. Reaction tubes containing the cell pellet were actively dried for 24 h at 80°C, passively dried for 24 h in a desiccator, and subsequently weighted. CDW determination was conducted in four technical replicates for each biological replicate.

### 2.5 Substrate analysis

Glc concentration was determined using an enzymatic glucose hexokinase assay (Glucose Hexokinase FS, DiaSys Diagnostic Systems GmbH, Holzheim, Germany). The procedure was conducted according to manufacturer instructions and automated on the liquid handling platform [61]. For calibration, a 5 g L^−1^ Glc stock solution was diluted to 2, 1.5, 1, 0.5, 0.25, 0.1, 0.05, and 0.025 g L^−1^ in three technical replicates. Cell‐free supernatants were appropriately diluted with 0.9% (w v^−1^) NaCl. Standards and samples were diluted 1:15 with the assay master mix and incubated for 6 min at room temperature. Calibration was conducted by linear regression of absorption at 340 nm with the given standard concentration. Glc determination was conducted in four technical replicates for each biological replicate.

### 2.6 DS‐FIA‐MS/MS

Single metabolite standards were prepared as 5 mM stocks in H_2_O and stored at −80°C. The calibration standard mixture was prepared as a 100 µM stock solution in 50% MeOH (v v^−1^) and stored at −80°C. The U‐^13^C^15^N labeled cell free amino acid mixture was diluted 1:4∙10^3^ with 50% MeOH (v v^−1^) to a final concentration of 1.25–16.25 µM and stored at −80°C.

For targeted analysis, a DS‐FIA‐MS/MS approach for metabolic profiling was used [59]. The calibration standard mixture was diluted with 50% MeOH (v v^−1^) in a sequential dilution series with 12 concentrations each. For the strong cation exchange method, a logarithmic dilution series of 50, 25, 10, 5, 2.5, 1, 0.5, 0.25, 0.1, 0.05, 0.025, and 0.01 µM was used. For the DS‐FIA methods, a linear dilution series of 16, 14, 12, 10, 8, 6, 4, 2, and 1 µM and subsequent logarithmic dilution series of 1, 0.5, 0.25, and 0.01 µM was applied. Samples were diluted using 50% MeOH (v v^−1^) in a sequential dilution series up to 1:2∙10^3^. Standards and samples were diluted 1:2 with a 1:4∙10^3^ diluted ^13^C^15^N labeled cell free amino acid mixture for isotope dilution mass spectrometry (IDMS). Standards and samples were distributed to V‐bottom microtiter plates, and subsequently sealed with self‐adhesive pierceable clear zone foil for automation (391–1264, VWR International GmbH, Darmstadt, Germany). Targeted analysis was conducted with four technical replicates for each biological replicate.

For semi‐targeted analysis, a DS‐FIA‐MS/MS approach for metabolic footprinting was used [60]. Samples were diluted using 50% MeOH (v v^−1^) in a sequential dilution series up to 1:10^3^. Quality control (QC) samples were prepared by sample pooling. Samples and QC samples were identically allocated to two V‐bottom microtiter plates which were subsequently sealed with self‐adhesive pierceable clear zone foil for automation (391–1264, VWR International GmbH, Darmstadt, Germany). Semi‐targeted analysis was conducted with four technical replicates for each biological replicate.

For mass spectrometry analysis, an Agilent 1100 system with an Agilent 1260 Infinity II Multisampler (Agilent Technologies, Waldbronn, Germany), coupled to an ESI‐QqQ (API4000, AB Sciex, Darmstadt, Germany) was used. Targeted analysis was conducted in MRM mode with a DS‐FIA‐MS/MS method for amino acid determination [59]. For verification of compounds with identical nominal mass and fragmentation pattern or respective fragment masses, a LC‐MS/MS method using a strong cation exchanger [59] was used for representative samples. For semi‐targeted analysis, a *C. glutamicum* genome model (KEGG: CGB) was used to develop an organism‐specific and time‐optimized DS‐FIA‐MS/MS method with 6 MRM packages, covering 224 metabolites with an analysis time of 1 min per package [60].

Instrument control and data acquisition was performed with Analyst 1.6.3 (ABSciex, Darmstadt, Germany).

### 2.7 Data processing

The extracted ion chromatograms of the MRM mode were automatically processed with the MQ4 algorithm of MultiQuant 3.0.3 (ABSciex, Darmstadt, Germany). Targeted metabolome analysis included normalization of standard and sample unlabeled analyte areas with labeled analyte areas by IDMS resulting in ^12^C^14^N/^13^C^15^N ratios. Calibration was performed by least squares approximation for linear regression of the ^12^C^14^N/^13^C^15^N peak area ratio with the corresponding concentration.

The source code and Jupyter notebook for semi‐targeted data evaluation can be found in the repository referenced in the supporting material section. For preliminary data processing of semi‐targeted metabolome experiments, signals were filtered by a signal‐to‐noise ratio >10. A low‐order nonlinear locally estimated smoothing function was fitted to the observed QC data with respect to sample injection order. Polynomials fitted to each subset of the data were based on weighted least squares. For smoothing parameter determination, leave‐one‐out cross‐validation was performed [62]. A correction function was obtained by fitting a cubic spline to the QC values predicted by the smoothing function. With respect to thresholding, metabolites were included for statistical analysis if they showed a relative standard deviation <20% for metabolites in the QC and missing data in the QC < 30% [63].

Univariate analysis of fold changes was performed with non‐parametric Kruskal–Wallis omnibus test [64] and parametric post‐hoc tests [65] for independent and dependent samples with a probability of error *α* = 0.05 and multi‐comparison correction based on Benjamini‐Hochberg (FDR) [66].

For multivariate analysis, missing data was mean imputed for values missing at random and half‐of‐the‐minimum imputed for values missing‐not‐at‐random [67]. Multivariate modeling based on principal component (PC) analysis and multi‐class partial least squares‐discriminant analysis (PLS‐DA) was conducted. Model evaluation and validation was based on stratified double 7‐fold cross‐validation in a pipeline with range scaling of the test and training predictor sets to avoid data leakage.

For hyperparameter determination, goodness‐of‐fit (R^2^X or R^2^Y) and goodness‐of‐prediction (Q^2^X or Q^2^Y) model performance indicator were used. R^2^Xcomp and R^2^Ycomp represent the explained variance of the sample‐feature matrix and response matrix by the corresponding PC or latent variable (LV). The optimal number of PCs or LVs was selected, if the goodness‐of‐prediction indicator did not increase by 5% if another PC/LC was added. If additional variance introduced by the cross‐validation procedure can be accounted for by a suitable model fit, a small difference between R^2^Y and Q^2^Y is expected. The PLS‐DA model was retrained with the optimal set of parameters and evaluated by bootstrap resampling (*r*  =  100) with replacement for percentile‐based confidence intervals [68, 69] of PLS regression coefficients and class specific variable importance in projection (VIP) scores [70].

For hierarchical cluster analysis (HCA), pairwise distances between observations were calculated based on the correlation distance metric, while hierarchical or agglomerative clustering was conducted with average linkage [71]. Normalization of HCA heatmaps was conducted for every feature by subtracting the minimum value and divide by its maximum value.

Over‐representation analysis (ORA) was conducted based on a hyperparametric test, a probability of error *α* = 0.05, and FDR multi‐comparison correction [66]. Pathway information was obtained by KEGG pathway maps [72, 73, 74] while normalized pathway impact for the pathway topology analysis (PTA) was calculated based on the directed graph metric betweenness centrality [75]. Supporting code and corresponding data files are available at https://github.com/JuBiotech/Supplement‐to‐Reiter‐et‐al.‐2023a (accessed on 09.09.2023) and DOI: 10.5281/zenodo.7657599.

## 3 RESULTS AND DISCUSSION

*C. glutamicum* is used, among other things, as a production organism for His. Due to the diverse applications of the essential amino acid and its increasing economic importance, bioprocess development for fermentative production of His using *C. glutamicum* is of utmost importance. However, research has primarily focused on rational strain development rather than fed‐batch process evaluation. To our knowledge, there has been no research that study fed‐batch cultivations of His producing *C. glutamicum* mutants utilizing a mineral salt medium. Therefore, an evaluation of fed‐batch processes for the microbial production of His by *C. glutamicum* with the CGXII mineral salt medium was performed in this study. This was supported by the use of high‐throughput methods for metabolite profiling to quantify product‐ and by‐product formation, as well as metabolic footprinting to study metabolic pattern in a timely manner.

### 3.1 Sensitivity analysis of media components using automated microscale cultivation platform

CGXII mineral salt medium is intended for batch cultivations and requires media optimization for the corresponding microbial strain and cultivation scale. To facilitate the media optimization or screening procedure, an automated cultivation platform utilizing small‐scale cultivation vessels was used to cope with the accruing experimental scope. Screening experiments conducted in small cultivation scale are generally not fully representative for subsequent process optimization experiments in laboratory‐scale. While the demand or consumption rate of a certain media component may still vary between scales, a limiting component in a functional small‐scale process is very likely a limiting component in a functional laboratory scale bioprocess. Therefore, the goal was to discover potential medium component limitations, that could have a significant influence during fed‐batch cultivation. The concentration of CGXII media components (NH_4_)_2_SO_4_, MgSO_4_·7 H_2_O, K_2_HPO_4_‐KH_2_PO_4_, CaCl_2_, biotin, PCA, trace element solution, and urea were reduced to 80, 60, 40, 20, and 10% in a one‐factor‐at‐a‐time approach. While a factorial experiment design would provide insights in substrate correlation for product formation optimization, its necessary parameter space optimization and potentially iterative methodology makes it less suitable for a high‐throughput screening approach. A one‐factor‐at‐a‐time approach is a suitable and simple design to identify metabolic bottlenecks, which are generally caused by single media components.

**FIGURE 1:**
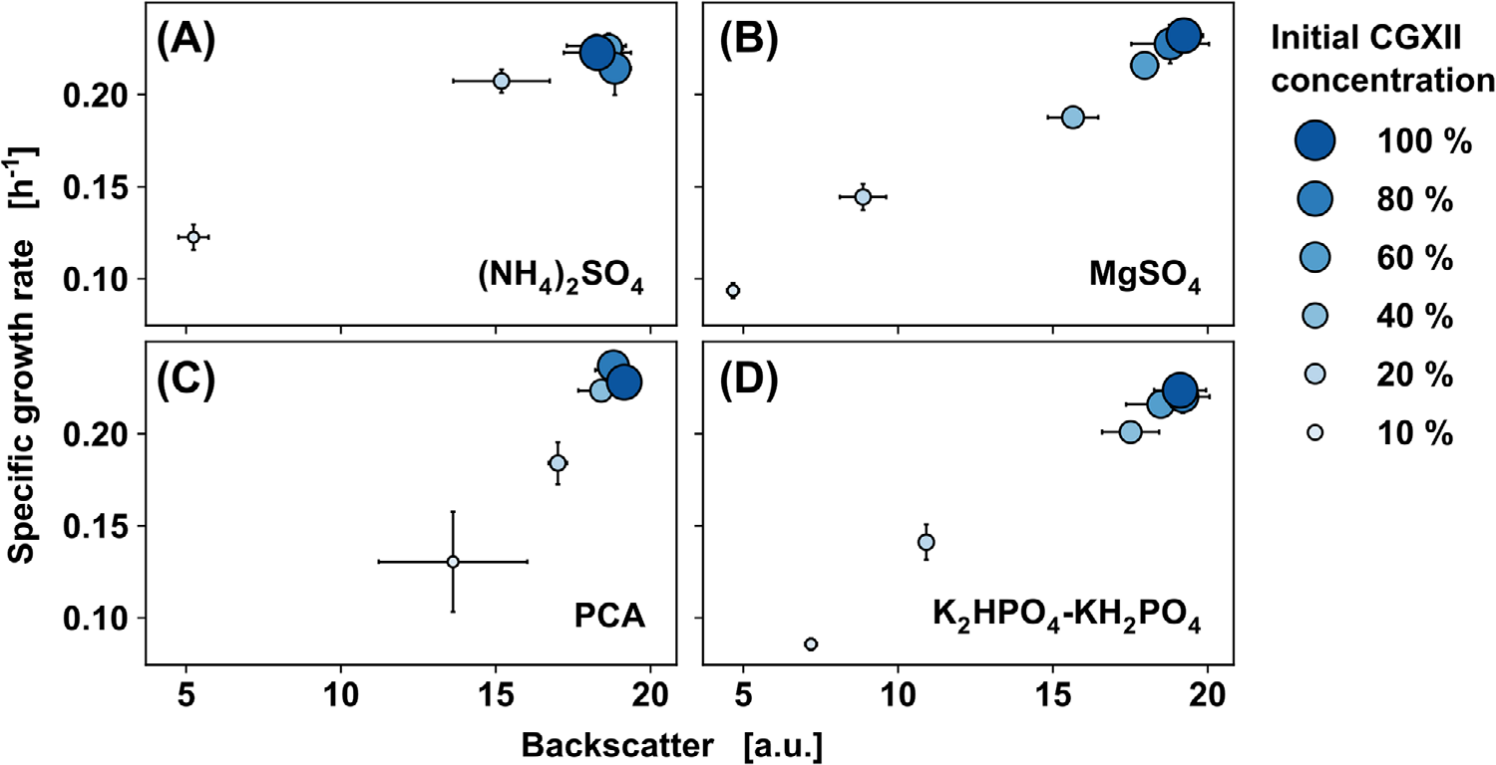
Sensitivity analysis for (A) (NH_4_)_2_SO_4_, (B) MgSO_4_, (C) PCA, and (D) K_2_HPO_4_‐KH_2_PO_4_ . CGXII medium components were reduced to 80, 60, 40, 20, and 10% of their initial concentration. The highest observed backscatter signal and specific growth rate are shown by mean values and standard deviations (n_biological_ = 3).

Cultivation was carried out in a BioLector with flower plate well design and online monitoring of pH and DO (cf. Figure S1 in the supporting information). Growth rate and final backscatter (BS) were used to evaluate effects of limiting media components on growth phenotype. The growth phenotype for (NH_4_)_2_SO_4_, MgSO_4_·7 H_2_O, PCA, and K_2_HPO_4_‐KH_2_PO_4_ is displayed in Figure 1A–D, showing a clear influence of these four media components with the growth rate. The influence of the remaining media components (biotin, CaCl_2_, trace element solution, urea) was very limited and is depicted in Figure S2 in the supporting information.

All cultivations show a sufficiently high DO for the different process conditions (cf. Figure S1 in the supporting information), which means that the observed influences on the cultivation are caused by the reduction of the media components. Lower concentrations of the CGXII media components (NH_4_)_2_SO_4_, MgSO_4_, PCA, and K_2_HPO_4_‐KH_2_PO_4_ show a clear influence on the growth rate and resulting biomass. While reduction of (NH_4_)_2_SO_4_ to 40% did not affect the growth phenotype, the reduction to 20% of the initial medium concentration resulted in approx. 15% lower maximum cell density as shown by BS, while further reduction to 10% of initial concentration, reduced final biomass and growth rate by approx. 70% and 50%, respectively. Since (NH_4_)_2_SO_4_ (cf. Figure 1A) is the primary nitrogen source in CGXII medium, a nitrogen limitation is implied.

Specific growth rate and cell density are limited in batch phase for MgSO_4_ (cf. Figure 1B) at 40% of initial medium concentration, with a somehow linear decrease for both parameters with decreasing MgSO_4_. Because (NH_4_)_2_SO_4_ provides the majority of the sulfur, the impact of limited MgSO_4_ on growth behavior is most likely related to an Mg^2+^ limitation. Mg^2+^ is required for many cellular processes, for example, certain enzymes as well as cell division. Furthermore, it has been observed that gram‐positive bacteria have a higher need for Mg^2+^ during cell division [76].

Reduction of PCA (cf. Figure 1C) resulted in a longer lag‐phase (data not shown) and cultivations with lower PCA concentration were not fully grown, before attaining final BS readings when the experiment was terminated after 20 h of cultivation time. PCA is aggressively metabolized by *C. glutamicum* at low cell densities during the first growth phase, but it is not required for biomass formation in subsequent phases [53].

The reduction of K_2_HPO_4_‐KH_2_PO_4_ (cf. Figure 1D) resulted in a similar growth phenotype as MgSO_4_ reduction (cf. Figure 1C), with reduced growth rate and final biomass concentration starting at 20% of the initial concentration. The response of *C. glutamicum* to phosphate deprivation was reported recently [77]. The transition from phosphate surplus to limiting conditions resulted in the expression of 25 distinct genes, all of which are involved in phosphorus absorption and phosphoester metabolism. A phosphate constraint is inferred since phosphate is a precursor for phosphor‐containing components and no alternate phosphate source was available in CGXII medium.

**FIGURE 2:**
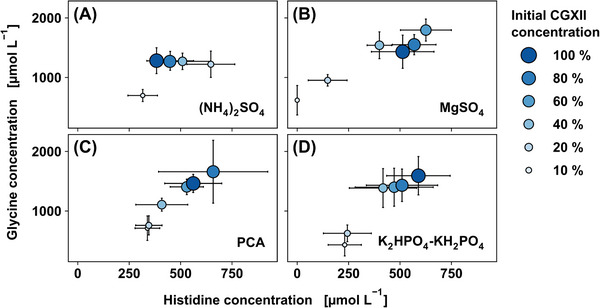
Sensitivity analysis for (A) (NH_4_)_2_SO_4_, (B) MgSO_4_, (C) PCA, and (D) K_2_HPO_4_‐KH_2_PO_4_ with respect to product formation. CGXII media components were reduced to 80, 60, 40, 20, and 10% of their initial concentration. The His and Gly titers are shown by mean values and standard deviations (n_biological_  =  3, n_technical_  =  4).

The amino acid determination using targeted DS‐FIA‐MS/MS was conducted to assess the metabolic phenotype in the form of main and by‐products. Figure 2 depicts the response of the metabolic phenotype in form of His and Gly titer for the reduction of (NH_4_)_2_SO_4_, MgSO_4_, PCA, and K_2_HPO_4_‐KH_2_PO_4_. The His and Gly titers for the remaining compound conditions (biotin, CaCl_2_, trace element solution, urea) are depicted in Figure S3 in the supporting information. As for the growth rate and final biomass, such parameters showed a very minor effect on His and Gly formation.

His and Gly formation (cf. Figure 2B–D) shows a similar trend to the growth phenotype (cf. Figure 1B–D) following reduction of MgSO_4_, PCA, and K_2_HPO_4_‐KH_2_PO_4_, implying growth‐related product formation. However, low concentrations of (NH_4_)_2_SO_4_ seem to benefit His formation while hampering growth (cf. Figures 1A and 2A). This represents a challenge with respect to growth coupled product formation. The reduction of MgSO_4_ to 60% resulted in the formation of Ala and Val (cf. Figure S4 in the supporting information), as well as decreased His production. Increased Ala and Val concentrations in the absence of cell growth were reported for *C. glutamicum* mutants after deleting the *aceE* gene, which encodes a component of the pyruvate dehydrogenase complex [78]. It was indicated, that the accumulating pyruvate in the stationary phase is channeled towards Val and Ala. One could speculate that the used strain carries a specific mutation around the pyruvate node from random mutagenesis. Furthermore, Mg^2+^ is important for activity of the HisG and HisI proteins in the His pathway [79].

A similar response was seen when K_2_HPO_4_‐KH_2_PO_4_ concentration was reduced to 20% of the initial medium component concentration (cf. Figure 2D). Although the buffering capacity of the medium was influenced by the variation of K_2_HPO_4_‐KH_2_PO_4_, the online pH data (see Figure S1 in the supporting information) show a sufficiently high buffering capacity by MOPS and therefore allow the exclusion of any pH effects on the product titer. Intracellular phosphate is a crucial component for energy metabolism and His pathway precursor formation. Since the His pathway is dependent on phosphorylated compounds such as phosphoribosylpyrophosphate and ATP, phosphate deficiency could show a direct impact on His production. Since the cumulative mass of K_2_HPO_4_‐KH_2_PO_4_ was reduced without varying the ratio, a preferred phosphate source cannot be identified in this case.

### 3.2 Process optimization for fed‐batch production of l‐histidine increases yield and volumetric productivity

**FIGURE 3:**
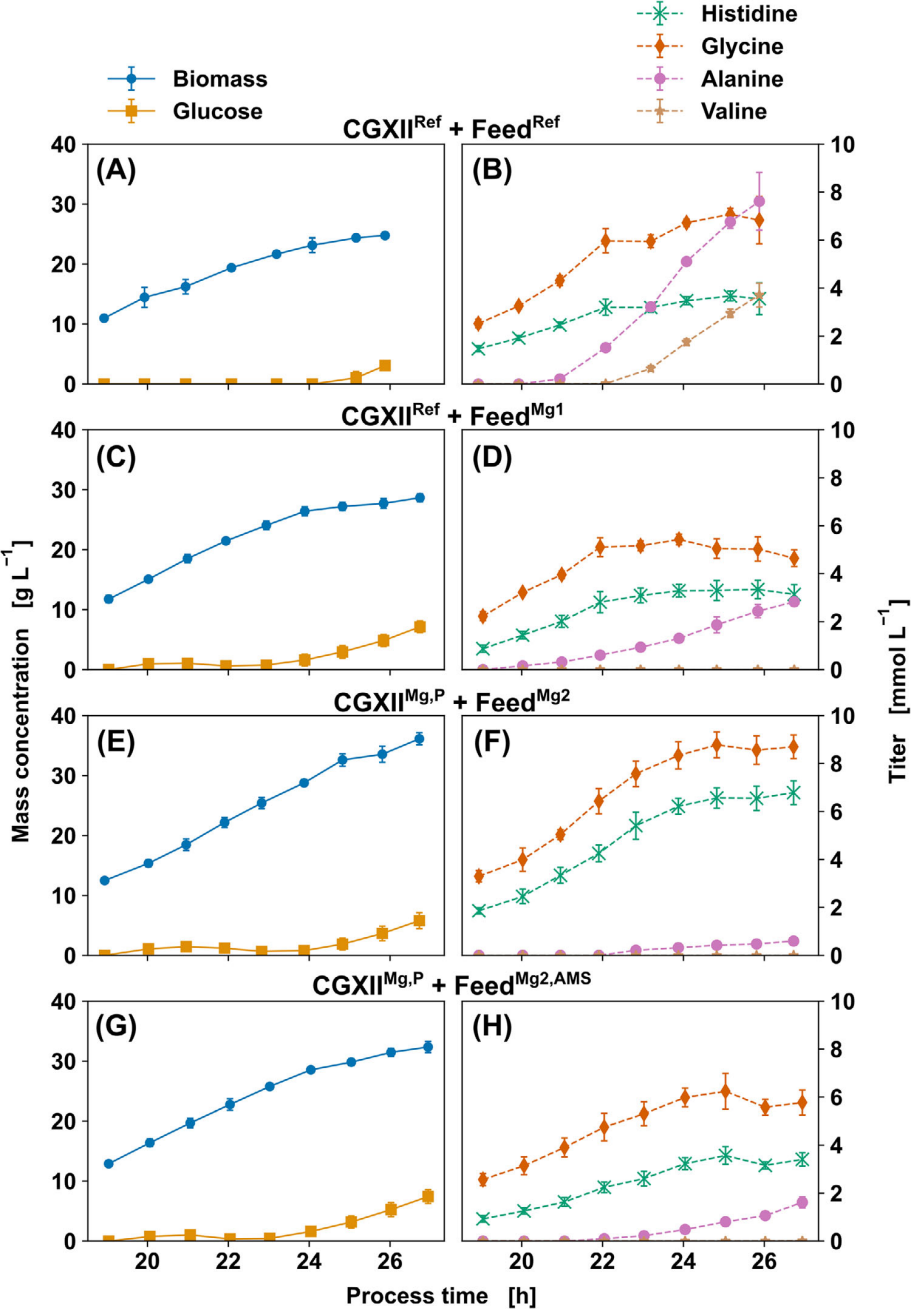
Process optimization of *C. glutamicum* for His production; displayed are the biomass and substrate mass concentrations (A,C,E,G) as well as product titer of His, Gly, Ala and Val (B,D,F,H) for the fed‐batch phase with mean and standard deviation (n_biological_  =  4, n_technical_  =  4). Initial batch cultivation was conducted in CGXII medium with 20 g L^−1^ glc for all cultivations. Fed‐batch conditions consist of a constant feed rate of 12.5 mL h^−1^ with 440 g L^−1^ glc. (A,B) Reference cultivation with CGXII^Ref^ and Feed^Ref^ with 440 g L^−1^ Glc; (C,D) CGXII^Ref^ and Feed^Mg1^ with 10 g L^−1^ MgSO_4_·7 H_2_O feed enrichment. (E,F) CGXII^Mg,P^ with 0.5 g L^−1^ MgSO_4_·7 H_2_O and 5 g L^−1^ K_2_HPO_4_ or 5 g L^−1^ KH_2_PO_4_ enrichment and Feed^Mg2^ with 7 g L^−1^ MgSO_4_·7 H_2_O feed enrichment. (G,H) CGXII^Mg,P^ and Feed^Mg2,AMS^ with 105 g L^−1^ (NH_4_)_2_SO_4_ feed enrichment.

Following the definition of bioprocess optimization targets, the generated information was used in the fed‐batch process to improve cultivation and feed medium. For this reason, a reference cultivation was established. Based on the reference cultivation and the previous sensitivity analysis of media components, base medium and feed were optimized for increased His production. For lab‐scale bioreactor cultivations (n_biological_ = 4), a two‐stage pre‐culture procedure was carried out. Batch cultivation was conducted with 20 g L^−1^ Glc in either basic CGXII (CGXII^Ref^) or enriched CGXII with 0.5 g L^−1^ MgSO_4_·7 H_2_O and 5 g L^−1^ K_2_HPO_4_ or 5 g L^−1^ KH_2_PO_4_ (CGXII^Mg,P^). During the fed‐batch phase, a constant feeding rate of 12.5 mL h^−1^ with 440 g L^−1^ Glc (Feed^Ref^) was used. For optimization, Feed^Ref^ was enriched with either 10 g L^−1^ MgSO_4_·7 H_2_O (Feed^Mg1^), 7 g L^−1^ MgSO_4_·7 H_2_O (Feed^Mg2^) or 7 g L^−1^ MgSO_4_·7 H_2_O and 105 g L^−1^ (NH_4_)_2_SO_4_ (Feed^Mg2,AMS^). Amino acid determination was conducted with the targeted DS‐FIA‐MS/MS method and IDMS with a runtime of 1 min per sample (n_technical_ = 4). The corresponding off‐line data for the fed‐batch phases is depicted in Figure 3. Key performance indicators for laboratory‐scale batch and fed‐batch cultures are listed in Table S1 in the supporting information along with the metrics for the sensitivity experiments from Section 3.1. With respect to the sensitivity experiments conducted in microtiter scale batch cultivations (CGXII^N^), His titer was increased from 0.59 ± 0.15 mM to 1.17 ± 0.19 mM after transferring the process to laboratory bioreactor scale (CGXII^Ref^). While microtiter scale cultivations were pH stabilized by MOPS buffer, lab‐scale bioreactor cultivations were pH controlled by acid and base titration. The histidine formation resulted in titration of H_3_PO_4_ (data not shown), a source of phosphate which was identified as a limiting component in Section 3.1.

The biomass concentration obtained by lab‐scale bioreactor batch cultivations (CGXII^Ref^, CGXII^Mg,P^) was increased from 11.38 to 24.77 g_CDW_ L^−1^, as expected, by utilizing fed‐batch mode with a constant Glc feed (CGXII^Ref^ + Feed^Ref^) (cf. Table S1 in the supporting information). In general, the batch cultivations resulted in higher biomass yields compared to fed‐batch processes which seems to indicate higher portion of product or by‐product formation in the growth limited fed‐batch phase (cf. Figure 3A). Growth‐coupled production plateaued after 22 h with a His titer of 3.56 ± 0.66 mM (cf. Figure 3B), implying some kind of additional limitation other than Glc for either cell growth or product formation. In addition, the ratio of His and Gly diverged over the course of the fed‐batch phase (cf. Figure 3B). Usually, a 1:1 ratio of His versus Gly could be expected if Gly formation is the only source of THF cofactor regeneration (cf. Figure S18 in the supporting information). The changing ratio itself, contradicts the mutual dependency of the THF regeneration and implies another source of THF for Gly formation. In addition, by‐products in form of Ala (7.61 ± 1.20 mM) and Val (3.71 ± 0.50 mM) accumulated within the first 21 h of cultivation (cf. Figure 3B), again pointing to Mg^2+^ or phosphate limitation as discussed in Section 3.1.

The feed enrichment with MgSO_4_ (CGXII^Ref^ + Feed^Mg1^) resulted in reduced Ala titer (2.83 ± 0.18 mM) and a suppression of Val production, which could indicate less pyruvate precursor availability (cf. Figure 3D). In addition, the His and Gly formation was aligned which indicates mutual THF regeneration for the fed‐batch phase suggesting a potential Mg^2+^ limitation which could be present for the His pathway enzymes during reference fed‐batch cultivation (CGXII^Ref^ + Feed^Ref^, cf. Figure 3B). However, while by‐product formation was reduced, the His product titer only reached 3.14 ± 0.4 mM, which was similar to the reference fed‐batch cultivation. Strikingly, additional medium enrichment with K_2_HPO_4_‐KH_2_PO_4_ (CGXII^Mg,P^ + Feed^Mg2^) resulted in the highest His titer of 6.79 ± 0.49 mM, the best product yield of 0.019 ± 0.001 mol_His_ mol_Glc_^−1^, almost no Ala formation (cf. Figure 3F and Table S1 in the supporting information) and no Val formation. Since phosphate is relevant for various cellular processes, several assumptions might be plausible. However, it can be stated, that additional inorganic phosphate promotes cell growth and therefore growth‐coupled production of His as displayed (cf. Figure 3E,F). Although the sensitivity analysis indicated a potential limitation for low concentrations of (NH_4_)_2_SO_4_, corresponding feed enrichment (CGXII^Mg,P^ + Feed^Mg2,AMS^) led to lower His titers and product selectivity (cf. Table S1 in the supporting information). The decrease in growth and production during the fed‐batch phase with (NH_4_)_2_SO_4_ (cf. Figure 3G,H) might be a result of substrate inhibition by either sulfate or ammonium, the latter being known for growth inhibition. As a conclusion, (NH_4_)_2_SO_4_ content in the base CGXII medium is sufficient for the batch and fed‐batch phases in this case.

By applying the optimized fed‐batch procedure (CGXII^Mg,P^ + CGXII^Mg2^), the His titer was improved by factor 5.8 and the product selectivity almost doubled with respect to lab‐scale bioreactor batch cultivation with basic CGXII medium (CGXII^Ref^). Furthermore, the His‐Gly ratio was aligned in the fed‐batch phase, representing the expected equimolar synthesis of the amino acids because of pathway linkage via THF recycling. Although bioprocess optimization was successful, missing metabolite information of a broader range of metabolites limits further strain or process development, especially to tackle the stagnation phase for His formation at approx. 24 h (cf. Figure 3B,D,F,G). Because the sensitivity analysis did not provide additional insights, a more comprehensive metabolic footprinting analysis to identify metabolites in the culture supernatant may be required. A semi‐targeted study of a larger metabolite set could yield such valuable information as identification of potential accumulating metabolic intermediates in His pathway for metabolic engineering.

### 3.3 Identification of optimization targets by metabolic footprinting

Given that simple product determination provides only a limited understanding of cell behavior, semi‐targeted metabolic footprinting may provide additional metabolic information. The identification and analysis of significantly altered metabolites or metabolic pathways allows for a thorough evaluation of the production system. The His biosynthesis route and associated metabolic pathways, including purine metabolism, are particularly valuable sources of information for future strain engineering and bioprocess development. Because the His pathway is highly branched, other metabolic pathways, in addition to those presently identified, may have an impact on His production.

The final batch and fed‐batch samples of the cultivation experiments conducted in Section 3.2 were examined using a metabolic footprinting approach based on the semi‐targeted DS‐FIA‐MS/MS method. Metabolites for semi‐targeted analysis were chosen based on their occurrence and precision in QC samples, as well as precision in cultivation samples, yielding 91 features or metabolites for multivariate analysis.

**FIGURE 4:**
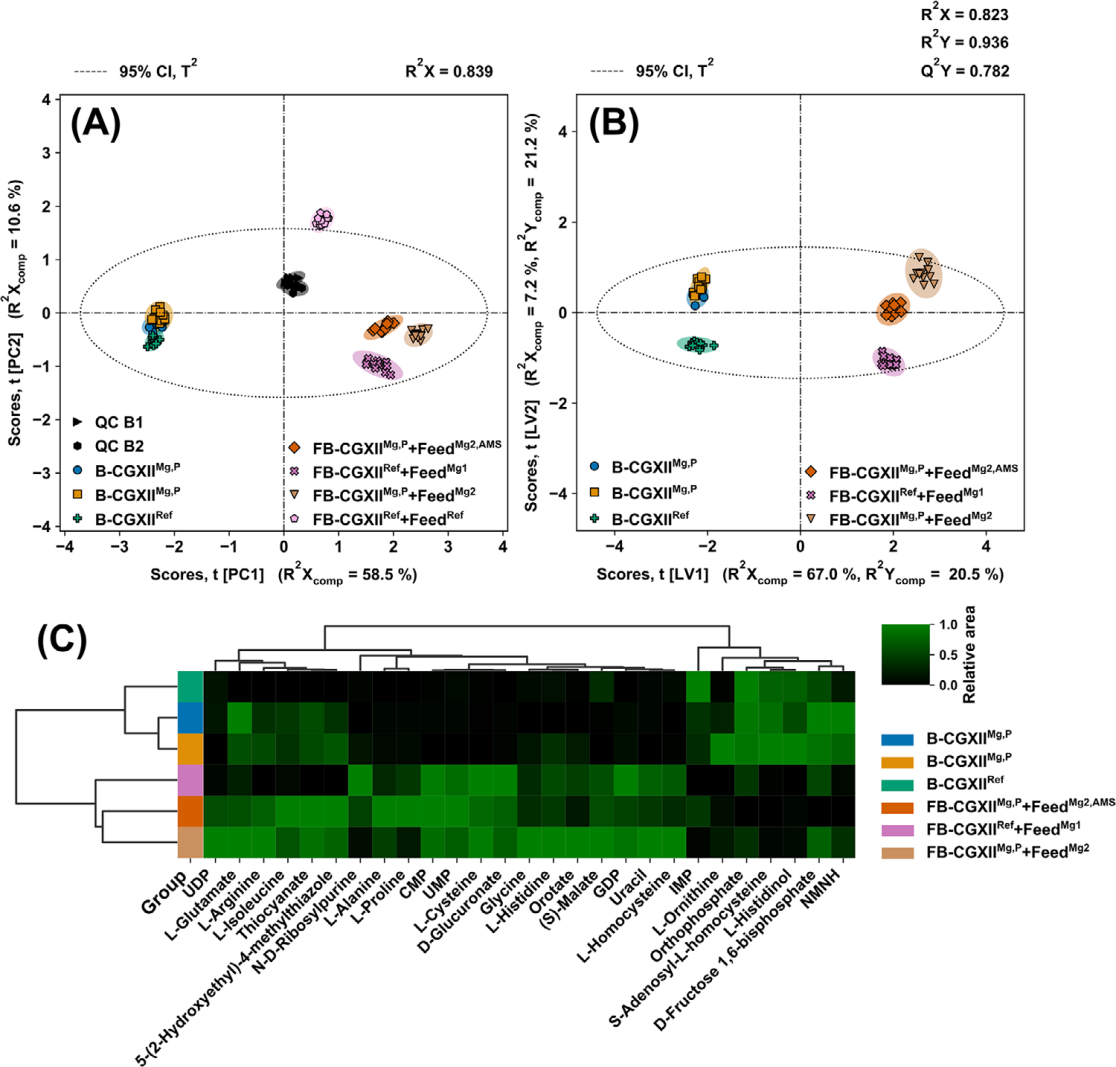
Semi‐targeted analysis of batch and fed‐batch cultivations; metabolic footprint of final batch (B‐CGXII^Ref^, B‐CGXII^Mg,P^) and fed‐batch (FB‐CGXII^Ref^ + Feef^Ref^, FB‐CGXII^Ref^+Feed^Mg1^, FB‐CGXII^Mg,P^ + Feed^Mg2^, FB‐CGXII^Mg,P^ + Feed^Mg2,AMS^) samples from bioprocess optimization (n_biological_  =  4, n_technical_  =  3). (A,B) Principal component analysis and PLS‐DA score plots of the first and second PC or LV with Hotelling's T^2^ ellipse for the 95% confidence interval (CI). (A) Principal component analysis scores plot with pooled QC samples in two analytical batches (QC B1, QC B2). (B) PLS‐DA scores plot. (C) HCA heatmap of the discriminating metabolites determined by class specific VIP scores.

The filtered data was assessed using PC analysis for QC (cf. Figure 4A) and PLS‐DA for variable selection and class discrimination (cf. Figure 4B). The goodness‐of‐prediction indicator (Q^2^X and Q^2^Y) was used to determine the ideal number of PCs and LVs (cf. Figure S5 in the supporting information). Class discriminating metabolites were subject to HCA for unsupervised clustering (Figure 4C). Because the CGXII^Mg,P^ + Feed^Mg2^ and CGXII^Mg,P^ + Feed^Mg2,AMS^ cultivations use the same batch medium, the CGXII^Mg,P^ class contains 8 biological and 24 technical replicates.

Close clustering of QC samples for two analytical batches (cf. Figure 4A) implies high inter‐batch repeatability, while tight clustering within each QC group indicates low intra‐batch variability. Final batch samples are clearly separated from final fed‐batch samples due to metabolites represented by the first PC. The influence of the features in the second PC causes the reference fed‐batch samples to fall outside Hotelling's T^2^ confidence interval. Although the position of the entire reference group would suggest an intriguing class, the related PLS‐DA variable selection for this class resulted in amino acids as the discriminating metabolites, with Gly and His excluded. The reference cultivation samples were therefore rejected for PLS‐DA due to low main and by‐product formation, as well as insufficient CGXII media component supply indicated in Section 3.2 (cf. Figure 4B). By excluding the outlier class in PLS‐DA, separation of batch and fed‐batch is acquired by metabolites represented by the second LV. The clear class separation of two batch and three fed‐batch procedures by visual inspection of the first two LV is validated by R^2^X of 0.823 to R^2^Y of 0.936, indicating that the PLS‐DA model adequately describes the data in the predictor and response matrices.

The discriminating metabolites of the remaining classes after VIP and beta coefficient thresholding are used to construct the HCA heatmap (cf. Figure 4C). The sample dendrogram on the vertical axis confirms class grouping in the PC analysis and PLS‐DA for batch and fed‐batch cultivations, respectively. By evaluating the feature dendrogram on the horizontal axis, the two main clusters responsible for distinguishing batch and fed‐batch samples are evident.

As expected, His and Gly are identified as discriminating metabolites between classes, accumulating from batch and fed‐batch cultivations. Furthermore, a decrease of orthophosphate from batch to fed‐batch is visible for all cultivations demonstrating the phosphate demand of the cells identified in Section 3.1. The heatmap also shows phosphorylated intracellular metabolites such as d‐fructose 1,6‐biphosphate, inosine monophosphate, and uridine monophosphate. The occurrence of such metabolites in cultivation supernatants is frequently observed and had already been studied [80]. It was concluded, that the metabolic state of the cell population correlates with the corresponding extracellular pools. Secretion of a typical intracellular metabolite was demonstrated under growth‐limited conditions with a Glc surplus, similar to the fed‐batch conditions described in Section 3.2 of this study.

The metabolites s‐adenosyl‐l‐homocysteine, l‐homocysteine, and Cys deplete or accumulate during fed‐batch phase, implying the participation of Cys and Met metabolism (KEGG: cgb00270) in His formation. Interestingly, l‐histidinol is identified as an accumulating precursor for His production in the batch phase, indicating *hisD* (cg2305) as a viable metabolic engineering target.

### 3.4 Investigation of process limitation by metabolic footprinting

Although an important precursor and interesting targets for further metabolic engineering were identified, the lack of time‐resolution prevents further exploration of the fed‐batch stagnation period beginning at 24 h as identified in Section 3.2. To avoid misinterpretation of additional limitation effects due to insufficient CGXII component concentrations, the optimized fed‐batch procedure (CGXII^Mg,P^ + Feed^Mg2^) was selected for further analysis. Because the discriminating footprints clearly show a shift from batch to fed‐batch end, a time resolved metabolic footprint (19, 21, 23, 25, and 27 h) was acquired (cf. Figure S6 for hyperparameter optimization in supporting information).

**FIGURE 5:**
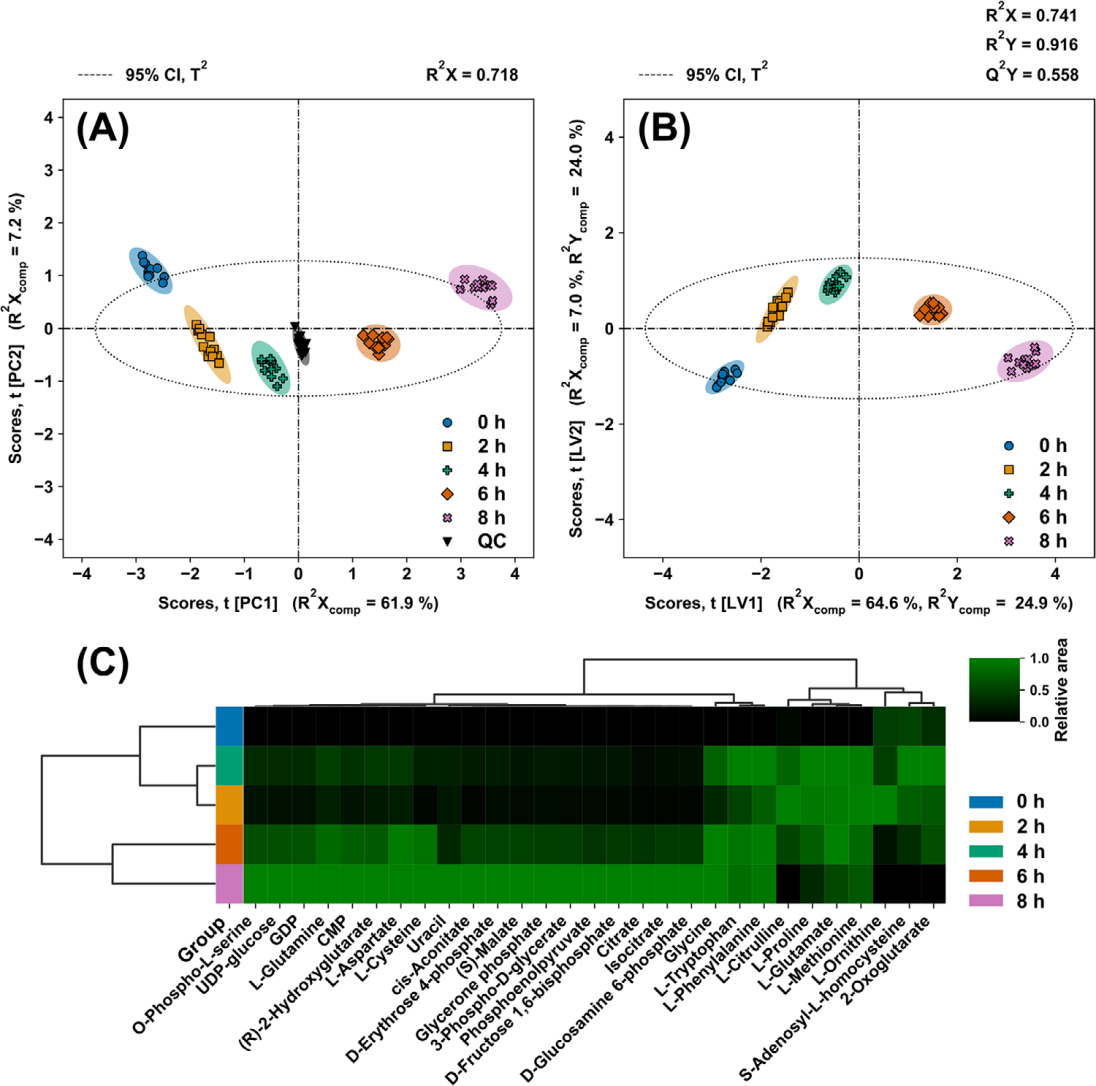
Semi‐targeted analysis of optimized fed‐batch cultivation; metabolic footprint of final batch (19 h) and fed‐batch (21, 23, 25, and 27 h) samples of FB‐CGXII^Mg,P^ + Feed^Mg2^ cultivation (n_biological_  =  3, n_technical_  =  4). (A,B) Principal component analysis and PLS‐DA score plots of the first and second PC or LV with Hotelling's T2 ellipse for the 95% confidence interval (CI). (A) Principal component analysis scores plot with pooled QC samples (QC). (B) PLS‐DA scores plot. (C) HCA heatmap of the discriminating metabolites determined by class specific VIP scores.

PC analysis scores for the first two components (cf. Figure 5A) demonstrate that QC samples are tightly clustered, indicating low intra‐batch variability. The metabolites represented by the first PC or LV are primarily responsible for sample or class discrimination (cf. Figure 5A,B). The time‐resolved footprint scores clearly show a metabolic shift during the fed‐batch phase. Tracking sample time point scores reveals an elbow between 23 and 25 h samples, indicating the transition phase, as seen in Figure 3 in Section 3.2 for His and Gly production. The PLS‐DA model, as depicted by the score plot of the first two LVs (cf. Figure 5B), was utilized for variable selection and the identification of discriminating metabolites. The related HCA heatmap of the discriminating metabolites (cf. Figure 5C) verifies variable selection by clustering non‐limited cultivation stages 19, 21, and 23 h, as well as limited cultivation stages 25 and 27 h on the vertical axis.

Accumulation of amino acids that were not included in targeted analysis, such as Glu and Met, is detected during the production phase (21 to 23 h). However, targeted analysis was performed with a dilution factor of 2·10^3^ instead of 1·10^3^. This indicates occurring concentrations <1 mM for these amino acids, falling below quantitation limits after dilution. However, the metabolites accumulating in the production phase (19 to 23 h) are depleting with the process entering the non‐forced limited state (23 to 27 h). This might be either due to dilution of constant metabolite levels at 23 h by titration volume or reconsumption by cells in the stationary state. An increase of typically intracellular metabolites such as phosphoenolpyruvate, glycerol phosphate, and d‐fructose 1,6‐bisphosphate is observed with the cell entering the limited cultivation phase. In conjunction, this seems to verify extended overflow metabolism for growth limited conditions with Glc surplus and additionally implies metabolite uptake in stationary cell state [80].

Overall, the time‐resolved semi‐targeted analysis seems to confirm the implied limitation phase in Section 3.2 by supplying a global limitation pattern expressed by the discriminating metabolic footprints. While feature analysis of these patterns may already provide single pathway targets, deducing a biological conclusion from single features is generally not possible due to the complexity of the pathways and cells. As a result, the study was expanded to include a pathway enrichment analysis of two distinct conditions.

### 3.5 Description of global pattern by metabolic pathway analysis

**FIGURE 6:**
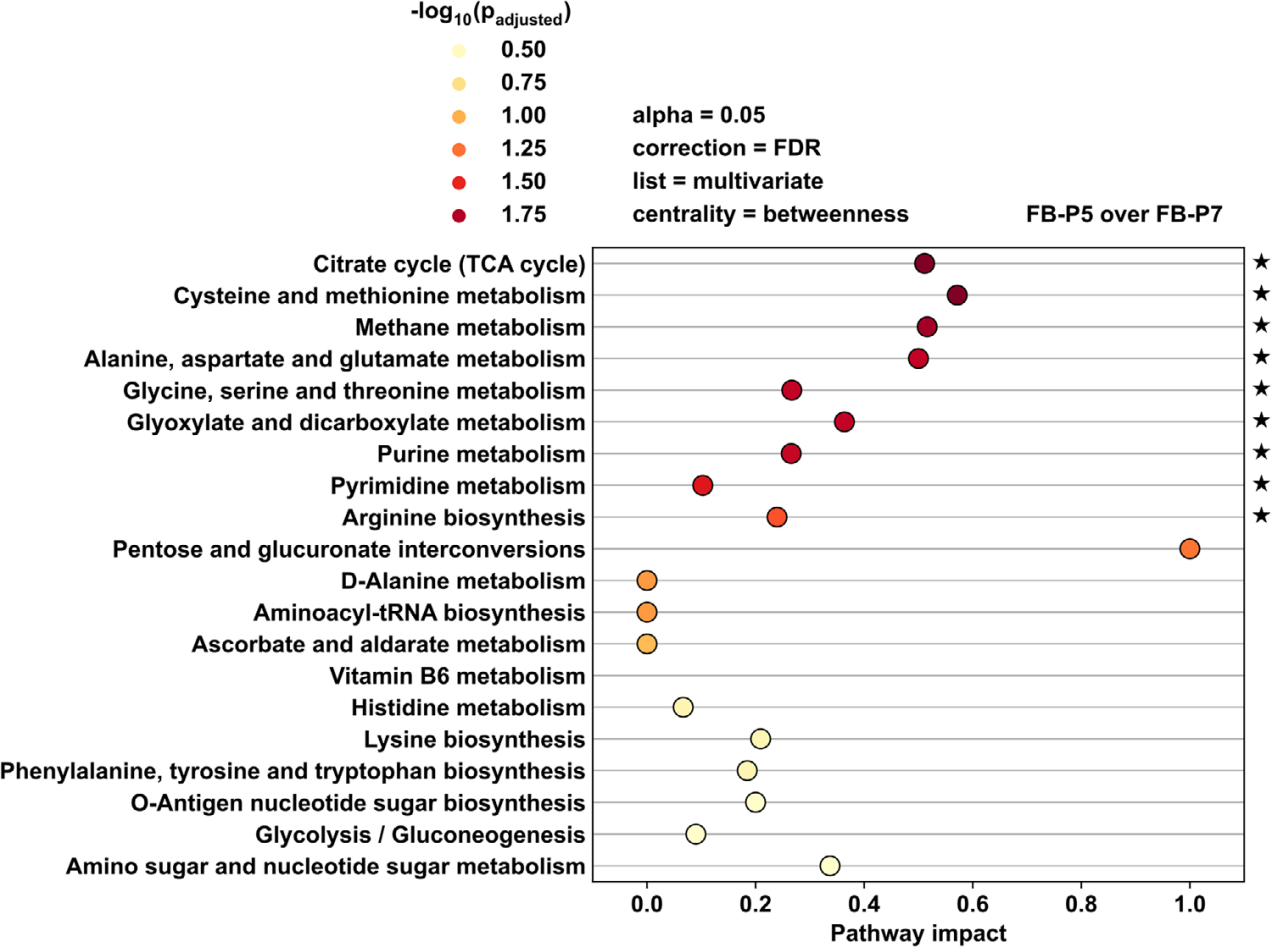
ORA and PTA; discriminating metabolites were identified by PLS‐DA variable selection; Conditions represent the non‐limited (23 h) and limited cultivation state (25 h), which were evaluated based on a hypergeometric test for pathway significance. Significantly changed pathways are indicated with a star. Displayed are the results for 20 of 55 KEGG pathway maps.

ORA and PTA were used to uncover significantly modified biological pathway expressions of the *C. glutamicum* mutant between two conditions for a given set of relevant metabolites. The samples after 23 and 25 h enclose the observed elbow (cf. Figure 5A,B) and represent two distinct conditions in form of forced Glc limitation or production phase and an additional limitation phase. Multivariate analysis based on a binary‐class PLS‐DA model (cf. Figure S7A,B in the supporting information) was conducted to provide a list of discriminating or interesting metabolites for pathway analysis. For PTA, KEGG pathway maps were employed, and pathway impact was estimated using the normalized betweenness‐centrality ratio (cf. Figure 6).

The ORA identified nine pathways based on the metabolite list acquired by multivariate analysis (cf. Figure 6). Pathway maps with relative node betweenness centrality scaling and significance coloring are available in Figures S8–S15 in the supporting information.

The citrate cycle, Cys and Met metabolism, methane metabolism which shares key components with glycolysis, as well as Ala, l‐aspartic acid, and Glu metabolism display relatively high pathway impact of approx. 0.5. The Cys and Met metabolism shows the highest pathway impact, owing to Met, S‐adenosyl‐L‐homocysteine, and s‐adenosyl‐l‐methionine (SAM) and their roles in information flow or betweenness centrality. The findings were validated by additional ORA and PTA analysis based on univariate analysis variable selection for 8 out of 9 pathways (cf. Figure S16–S17 in the supporting information), indicating that the Cys and Met metabolism responded to the conditions observed during cultivation.

The identified key metabolites Met, s‐adenosyl‐l‐homocysteine, and s‐adenosyl‐l‐methionine are all part of the Met cycle linked to l‐homocysteine. The methyltransferase *metH* catalyzes the reaction from l‐homocysteine to Met utilizing 5‐methyl‐tetrahydrofolate (5‐mTHF) [81], implying that a THF derivative is regenerated by *metH* (cf. Figure S18 in the supporting information).

*metF* can convert 10‐formyl‐tetrahydrofolate (10‐fTHF) to 5‐mTHF, which acts as a cofactor in the Met cycle conversion from l‐homocystein to Met catalyzed by *metH*. Since 5‐mTHF is required for Met cycle activity, it is safe to assume that a fraction of the 5,10‐mTHF is converted by *metF* rather than *folD* in the folate cycle (cf. Figure S18 in the supporting information). THF is available for Gly production as a result of Met formation by *metH*, resulting in non‐equimolar His‐Gly ratios. This would provide an indirect link between the reported His production pathways and the Met pathway.

Met and s‐adenosyl‐l‐homocysteine accumulated in the production phase, indicating potential bottleneck reactions as part of the Met THF regeneration cycle. s‐adenosyl‐l‐methionine, which has regulatory effects in Met biosynthesis of *C. glutamicum* [82], accumulated during the non‐forced limitation phase. Extracellular accumulation of s‐adenosyl‐l‐methionine implies some kind of overflow metabolism due to intracellular accumulation of such, which would inhibit the Met cycle for THF regeneration.

However, the use of a rapid and semi‐targeted approach based on DS‐FIA‐MS/MS enabled the validation of the limitation phase described in Section 3.2 based on the metabolic footprint. l‐histidinol, an accumulating precursor for His biosynthesis, was found as a discriminating metabolite for batch and fed‐batch experiments. Time‐resolved metabolic footprinting revealed distinguishing and variable intermediates for the optimized fed‐batch process. ORA and PTA identified the Cys and Met metabolism pathway or Met pathway as a significantly changed metabolite set between the forced and non‐forced limitation phases during fed‐batch cultivation. The intriguing Met pathway intermediates are components of the regulated Met cycle, which is also involved in THF regeneration.

## 4 CONCLUDING REMARKS

The presented study demonstrates a bioprocess optimization workflow for *C. glutamicum* in CGXII minimal media for His production. Screening of several media conditions was conducted utilizing a microscale and highly parallelized bioreactor system. The integration of such system into a liquid handling robot allowed for automated sampling and sample processing procedures for subsequent multicomponent analysis. With the application of a column‐free and targeted DS‐FIA‐MS/MS approach, main and by‐product amino acid formation was quantified with an analysis time of 1 min / sample. The information of the high‐throughput experiments was used for lab‐scale bioreactor bioprocess optimization. By‐product information and latent patterns were identified using a rapid semi‐targeted DS‐FIA‐MS/MS approach for metabolic footprinting based on a *C. glutamicum* genome model.

At first, high‐throughput cultivation of His producing *C. glutamicum* in varying medium compositions with decreasing concentrations of a specific CGXII component was performed to induce medium limitations. The analysis of growth and metabolic phenotypes enabled the identification of medium limitations for decreasing MgSO_4_, K_2_HPO_4_‐KH_2_PO_4_, and (NH_4_)_2_SO_4_ availability. His and Gly production was shown to be correlated and growth coupled, which further provided input for lab‐scale bioreactor cultivations.

The approach for producing His with *C. glutamicum* in CGXII mineral salt medium was transferred to laboratory bioreactor scale for fed‐batch cultivation. Based on the results of small‐scale experiments, process optimization was carried out with MgSO_4_ and K_2_HPO_4_‐KH_2_PO_4_ enrichment of the base and feed medium. The addition of MgSO_4_ aligned the initially observed diverging formation of His and Gly, implying that non‐equimolar production of His and Gly is rooted prior to the fed‐batch phase. The improved lab‐scale bioreactor fed‐batch process improved the His titer by factor 5.8 to 6.79 ± 0.49 mM, while increasing the product yield to 0.019 ± 0.001 mol_His_ mol_Glc_^−1^. Reported yields for rationally designed His‐producing *C. glutamicum* mutants in literature are 4.9 times higher (0.093 ± 0.003 mol_His_ mol_Glc_^−1^) [40]. However, the improvement achieved in this study was solely based on process development and medium optimization of CGXII, which for this process is not reported so far. It is also noteworthy that the used strain in this study was acquired by random mutagenesis, which only represents a basis for further rational strain engineering. It can be assumed, that the optimization conducted in this study is also applicable to rationally designed strains.

The identification of latent patterns and relevant features for future work was facilitated by high‐throughput metabolic footprinting using the semi‐targeted DS‐FIA‐MS/MS method. The addition of a time‐resolved metabolic footprinting approach for the optimized procedure corroborated the observed non‐forced limiting phase by a larger selection of metabolites and strengthened the notion of Met pathway participation. While feature analysis revealed AICAR and l‐histidinol as an additional accumulating precursor, ORA and PTA allowed the Met biosynthesis to be identified as a changed set of metabolites between the forced and non‐forced limitation phases, indirectly connecting the commonly described purine and Gly pathways for His formation in *C. glutamicum* to the Met pathway.

With respect to bioprocess optimization, this study represents the first reported process of His production with *C. glutamicum* in laboratory fed‐batch cultivations based on CGXII mineral salt medium. Furthermore, potential optimization targets were identified by hypothesis generating and semi‐targeted DS‐FIA‐MS/MS indicating Met biosynthesis participation in His formation, which is not reported so far.

Although the current screening approach implied Met biosynthesis participation, future work should include metabolic fingerprinting as a supporting methodology. The availability of intracellular metabolite levels, as well as the detection of potential pathway bottlenecks caused by limiting enzyme activity and intermediate accumulation, would be a distinct advantage. Furthermore, due to the quenching and extraction methodology, a fingerprint would provide a true snapshot of the intracellular metabolome for a given sampling time, whereas a footprint may represent a delayed response of the cell due to transport effects. Nonetheless, the acquisition of the metabolic fingerprint as an intracellular measurement significantly increases operator time and expense.

The presented workflow utilized high‐throughput technologies to identify media optimization targets in the context of bioprocess development and metabolic engineering. The successfully proven workflow is a valuable tool for initial screening procedures because the methodologies are either highly automated or time and cost effective. This clearly implies the applicability to either different organisms, media compositions, or process conditions.

## NOMENCLATURE

**Table:** 

BS	[‐]	backscatter
CDW	[g L^−1^]	cell dry weight
DO	[%]	dissolved oxygen
n	[‐]	number of samples
OD_600_	[‐]	optical density at 600 nm
Q^2^X	[‐]	goodness‐of‐prediction (predictor)
Q^2^Y	[‐]	goodness‐of‐prediction (response)
r	[‐]	number of bootstrapping samples
R^2^X	[‐]	goodness‐of‐fit (predictor)
R^2^Y	[‐]	goodness‐of‐fit (response)
R^2^X_comp_	[‐]	goodness‐of‐fit, single component (predictor)
R^2^Y_comp_	[‐]	goodness‐of‐fit, single component (response)
VIP score	[‐]	variable importance in projection scores
v v^−1^	[L L^−1^%]	volume fraction
w w^−1^	[g g^−1^%]	weight fraction
Greek symbols		
*α*	[‐]	probability of error
µ	[h^−1^]	specific growth rate
Indices		
AA	[‐]	amino acid
Glc	[‐]	d‐glucose
Biological	[‐]	biological replicate
Technical	[‐]	technical replicate

## CONFLICT OF INTEREST STATEMENT

The authors declare no conflicts of interest.

## Supporting information

Supporting Information

Supporting Information
