# Supplementary material for: Rapid exometabolome footprinting combined with multivariate statistics: A powerful tool for bioprocess optimization
Source: Eng Life Sci. 2024 Mar 5;25(2):2300222. doi: 10.1002/elsc.202300222 (PMC11842285; doi:10.1002/elsc.202300222)
Supplement: Supplementary file 1 — Supporting Information [file ELSC-25-2300222-s001.pdf]

Supporting information

Research Article

Rapid exometabolome footprinting combined with multivariate statistics: a powerful tool for bioprocess optimization

Alexander Reiter[1,2]

Lars Wesseling[1]

Wolfgang Wiechert[1,3]

Marco Oldiges[1,2]

[1] Institute of Bio- and Geosciences, IBG-1: Biotechnology, Forschungszentrum Jülich GmbH, Jülich, Germany

[2] Institute of Biotechnology, RWTH Aachen University, Aachen, Germany

[3] Computational Systems Biotechnology, RWTH Aachen University, Aachen, Germany

Correspondence: Prof. Dr. Marco Oldiges (m.oldiges@fz-juelich.de). Institute of Bio- and Geosciences, IBG-1: Biotechnology, Forschungszentrum Jülich GmbH, Wilhelm-Johnen-Straße, 52425 Jülich, Germany

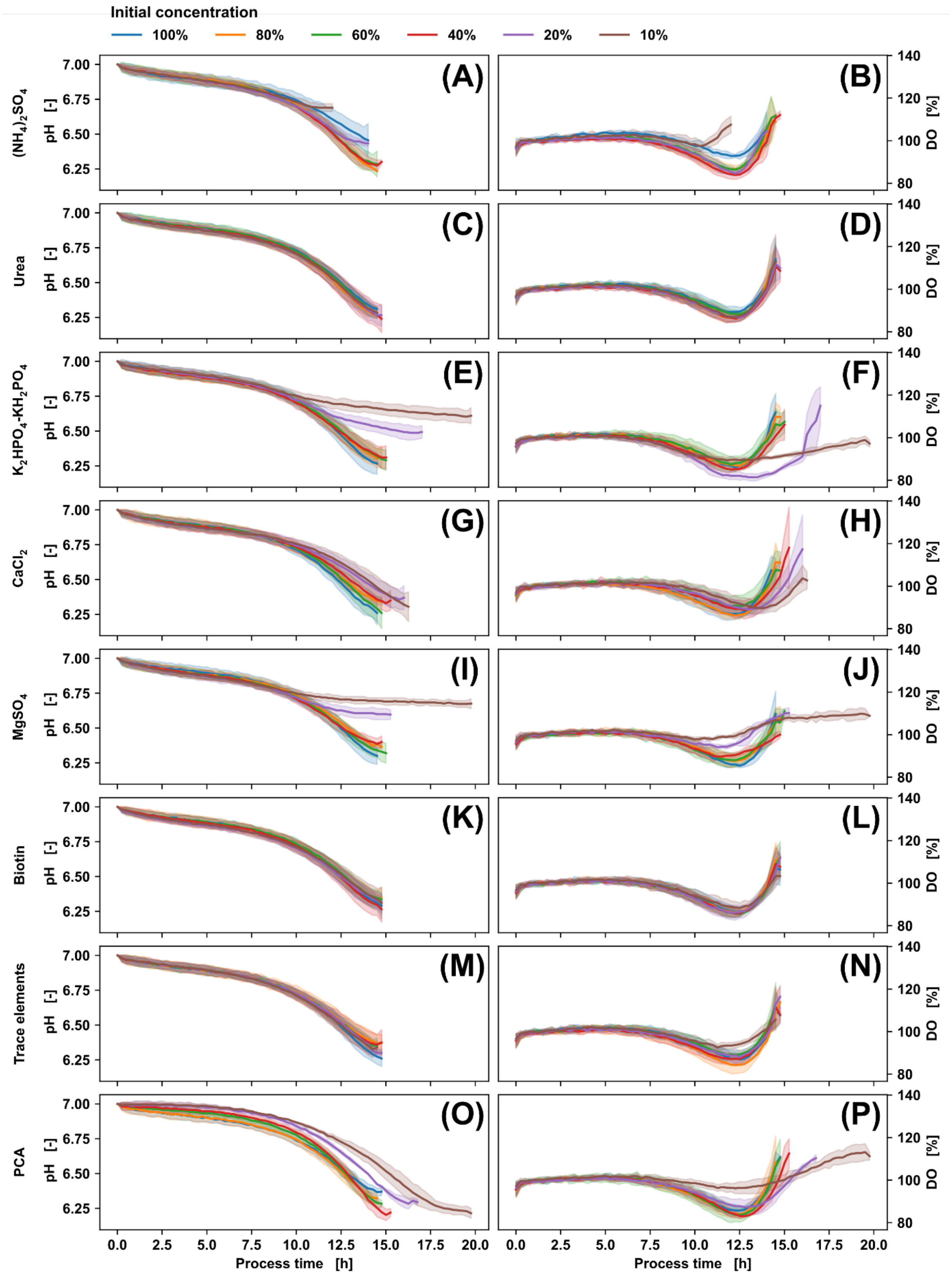

Figure S1: Sensitivity analysis cultivation data; pH and dissolved oxygen for (A, B) $(NH_4)_2SO_4$, (C, D) urea, (E, F) K_2HPO_4-KH_2PO_4, (G, H) $CaCl_2$, (I, J) $MgSO_4$, (K, L) biotin, (M, N) trace elements, (O, P) protocatechuic acid (PCA). CGXII medium components were reduced to 80, 60, 40, 20, and 10 % of their initial concentration. Online pH and DO are shown by mean values and standard deviations ($n_{biological} = 3$)

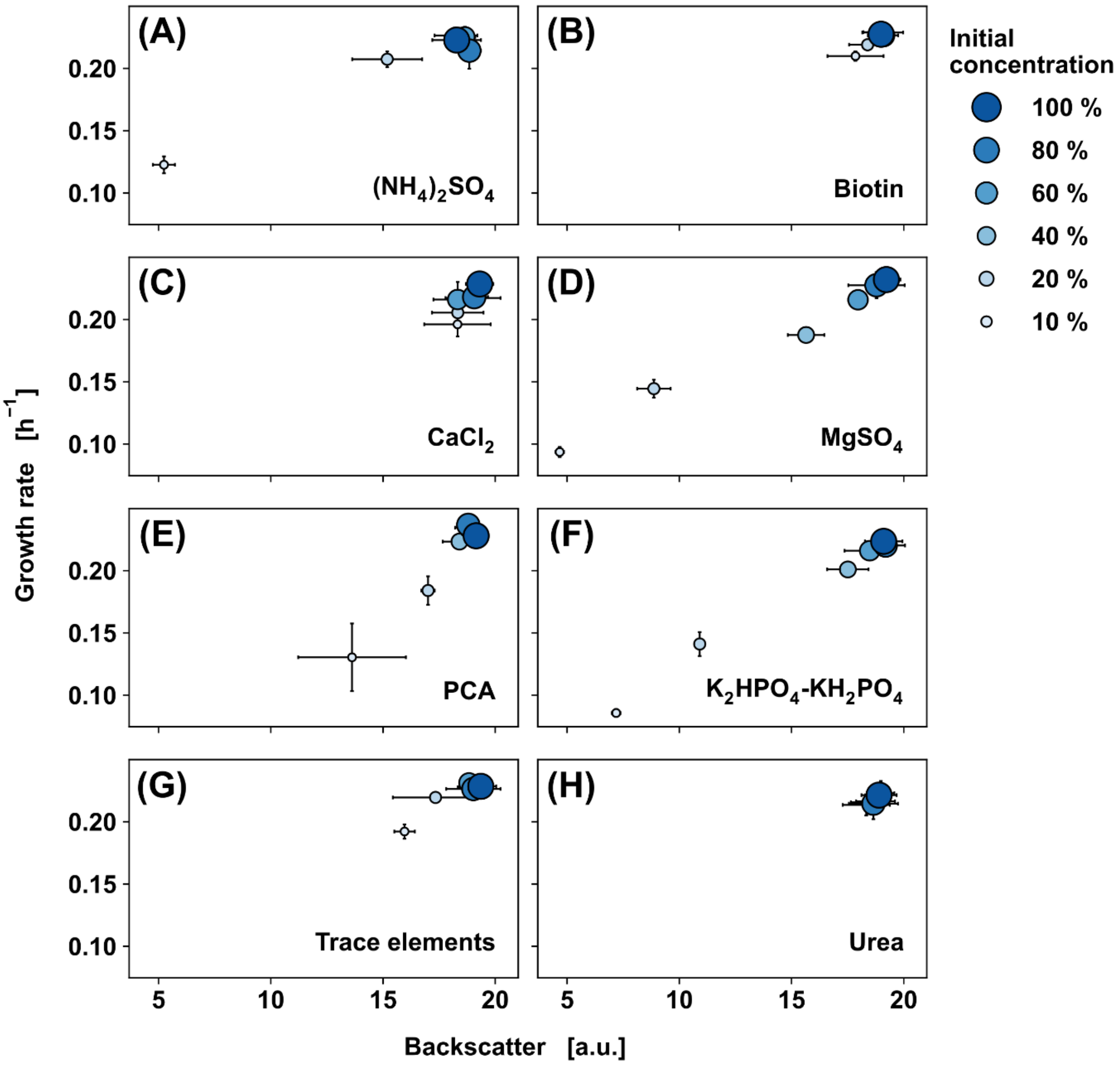

Figure S2: Sensitivity analysis with respect to cell growth; sensitivity analysis for (A) $(NH_4)_2SO_4$, (B) biotin, (C) $CaCl_2$, (D) $MgSO_4$, (E) protocatechuic acid (PCA), (F) K_2HPO_4-KH_2PO_4, (G) trace element solution and (H) urea. CGXII medium components were reduced to 80, 60, 40, 20, and 10 % of their initial concentration. The highest observed backscatter signal and specific growth rate are shown by mean values and standard deviations ($n_{biological} = 3$, $n_{technical} = 4$)

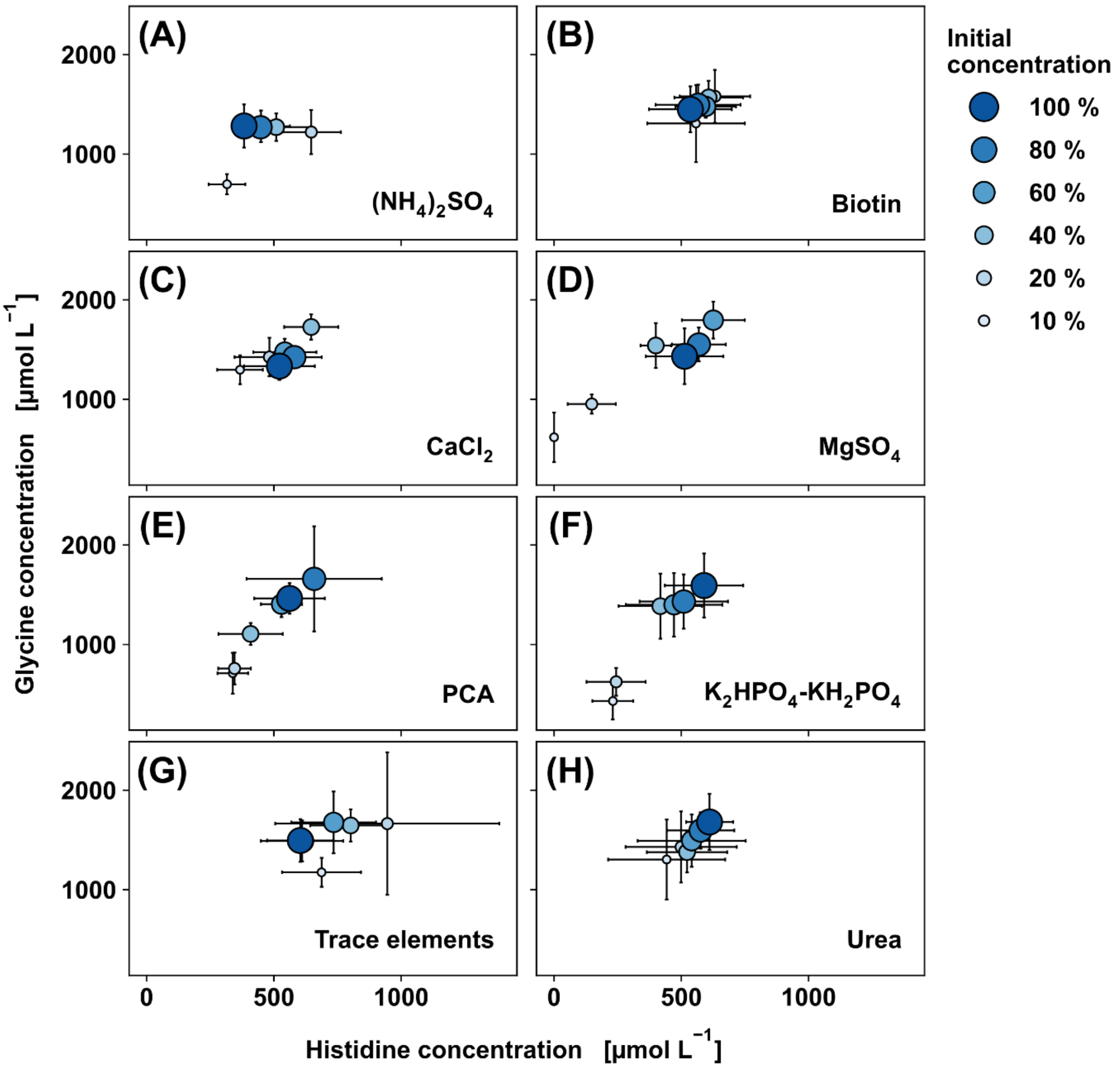

Figure S3: Sensitivity analysis with respect to L-histidine and L-glycine formation; sensitivity analysis for (A) $(NH_4)_2SO_4$, (B) biotin, (C) $CaCl_2$, (D) $MgSO_4$, (E) protocatechuic acid (PCA), (F) K_2HPO_4-KH_2PO_4, (G) trace element solution and (H) urea. CGXII medium components were reduced to 80, 60, 40, 20, and 10 % of their initial concentration. The L-histidine and L-glycine titer are shown by mean values and standard deviations ($n_{biological} = 3$, $n_{technical} = 4$)

Table S1: Key performance indicator of process optimization; concentration, volumetric productivity and yield for biomass and amino acids; $CGXII^{N}$: 200 mM MOPS buffered CGXII with 20 g L^{-1} Glc; $CGXII^{Ref}$: CGXII with 20 g L^{-1} Glc; $CGXII^{Mg,P}$: $CGXII^{Ref}$ with 0.5 g L^{-1} $MgSO_4 \cdot 7\ H_2O$ and 5 g L^{-1} K_2HPO_4 or 5 g L^{-1} KH_2PO_4 enrichment; $Feed^{Ref}$: Constant feed rate of 12.5 mL h^{-1} with 440 g L^{-1} Glc; $Feed^{Mg1}$: $Feed^{Ref}$ with 10 g L^{-1} $MgSO_4 \cdot 7\ H_2O$ feed enrichment; $Feed^{Mg2}$: $Feed^{Ref}$ with 7 g L^{-1} $MgSO_4 \cdot 7\ H_2O$ feed enrichment; $Feed^{Mg2,AMS}$: $Feed^{Mg2}$ with 105 g L^{-1} $(NH_4)_2SO_4$ feed enrichment;

**Key performance indicator**	**Analyte**	**Sensitivity**	**Optimization**					
		**$CGXII^{N}$**	**$CGXII^{Ref}$**	**$CGXII^{Mg,P}$**	**$CGXII^{Ref}$ + $Feed^{Ref}$**	**$CGXII^{Ref}$ + $Feed^{Mg1}$**	**$CGXII^{Mg,P}$ + $Feed^{Mg2}$**	**$CGXII^{Mg,P}$ + $Feed^{Mg2,AMS}$**
Concentration [g_{CDW} L^{-1}]	CDW	-	11.38 ± 0.65	12.7 ± 0.48	24.77 ± 0.14	28.67 ± 0.64	36.13 ± 0.96	32.35 ± 0.92
Yield [g_{CDW} g_{Glc}]	CDW	-	0.58 ± 0.04	0.64 ± 0.02	0.43 ± 0.01	0.44 ± 0.01	0.55 ± 0.01	0.49 ± 0.01
Titer [$mmol_{AA}$ L^{-1}]	Ala	0	0	0	7.61 ± 1.2	2.83 ± 0.18	0.6 ± 0.12	1.62 ± 0.23
	Gly	1.59 ± 0.32	2.37 ± 0.25	2.93 ± 0.34	6.83 ± 0.98	4.64 ± 0.34	8.7 ± 0.48	5.78 ± 0.52
	His	0.59 ± 0.15	1.17 ± 0.19	1.39 ± 0.18	3.56 ± 0.66	3.14 ± 0.4	6.79 ± 0.49	3.41 ± 0.28
	Lys	0.76 ± 0.18	1.69 ± 0.3	0.81 ± 0.24	14.73 ± 0.97	9.58 ± 0.99	11.52 ± 0.51	7.12 ± 0.47
	Val	0	0	0	3.71 ± 0.5	0	0	0.02 ± 0.04
Volumetric productivity [μmol_{AA} L^{-1} h^{-1}]	Ala	4.4 ± 3.17	0	0.07 ± 0.3	294.2 ± 46.3	105.8 ± 6.9	22.3 ± 4.4	60.0 ± 8.4
	Gly	108.3 ± 20.9	125.1 ± 13.4	153.83 ± 18.0	263.9 ± 38.0	173.7 ± 12.8	325.4 ± 18.0	214.4 ± 19.3
	His	40.1 ± 10.4	61.8 ± 10.3	73.11 ± 10.0	137.5 ± 25.6	117.4 ± 15.1	253.8 ± 18.5	126.5 ± 10.4
	Lys	51.39 ± 12.17	88.9 ± 16.0	42.55 ± 12.9	569.2 ± 37.3	358.2 ± 36.9	430.8 ± 18.9	264.5 ± 17.5
	Val	0	0	0	143.5 ± 19.1	0	0	0.6 ± 1.5
Yield [mol_{AA} mol_{Glc}]	Ala	-	0	0	0.024 ± 0.004	0.008 ± 0.001	0.002 ± 0.001	0.004 ± 0.001
	Gly	-	0.022 ± 0.002	0.027 ± 0.003	0.021 ± 0.003	0.013 ± 0.001	0.024 ± 0.001	0.016 ± 0.001
	His	-	0.011 ± 0.002	0.013 ± 0.002	0.011 ± 0.002	0.009 ± 0.001	0.019 ± 0.001	0.009 ± 0.001
	Lys	-	0.016 ± 0.003	0.008 ± 0.002	0.046 ± 0.003	0.027 ± 0.003	0.031 ± 0	0.019 ± 0.001
	Val	-	0	0	0.012 ± 0.001	0	0	0

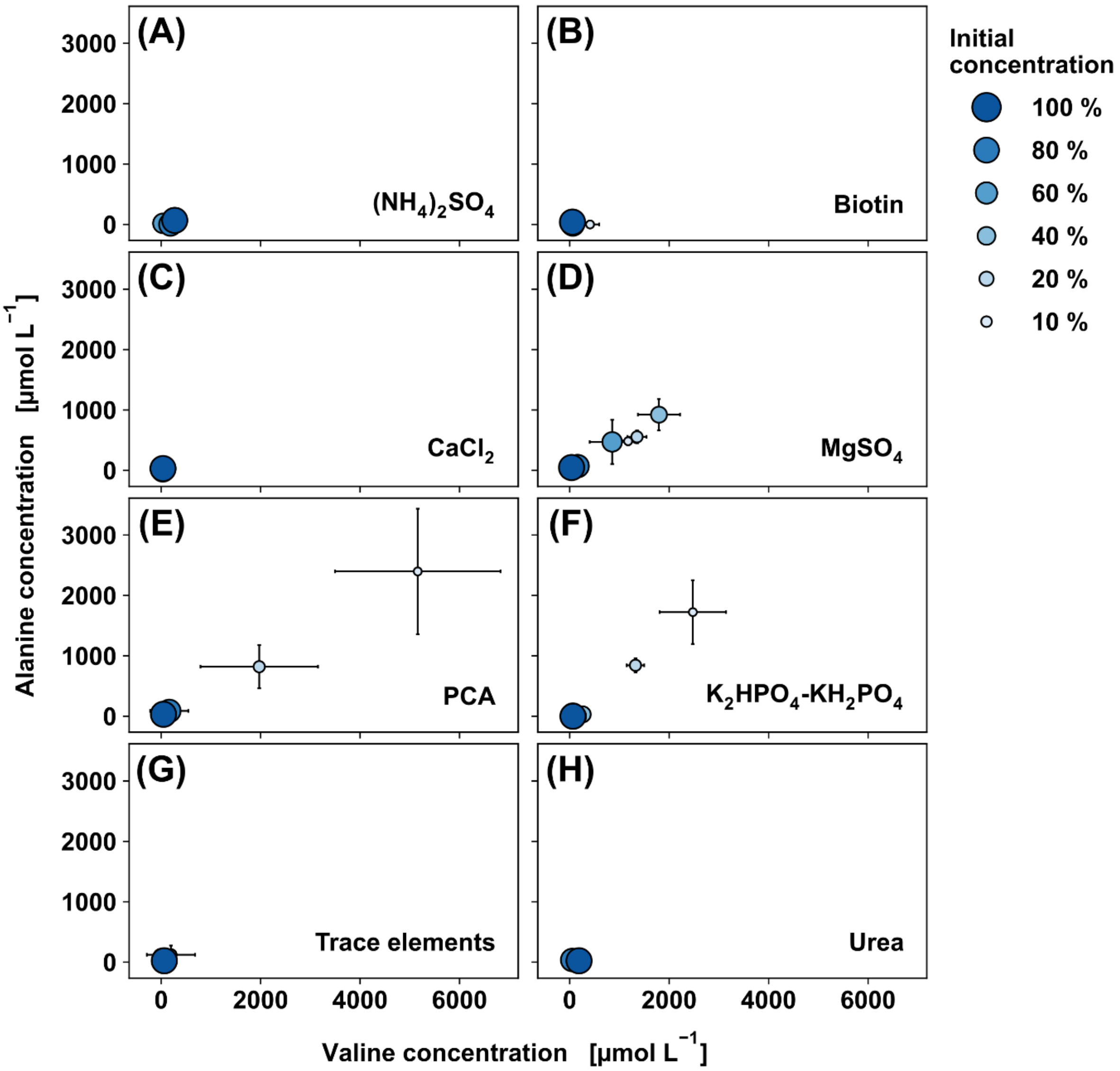

Figure S4: Sensitivity analysis with respect to L-alanine and L-valine formation; Sensitivity analysis for (A) $(NH_4)_2SO_4$, (B) biotin, (C) $CaCl_2$, (D) $MgSO_4$, (E) protocatechuic acid (PCA), (F) K_2HPO_4-KH_2PO_4, (G) trace element solution and (H) urea. CGXII medium components were reduced to 80, 60, 40, 20, and 10 % of their initial concentration. The L-alanine and L-valine titer are shown by mean values and standard deviations ($n_{biological} = 3$, $n_{technical} = 4$)

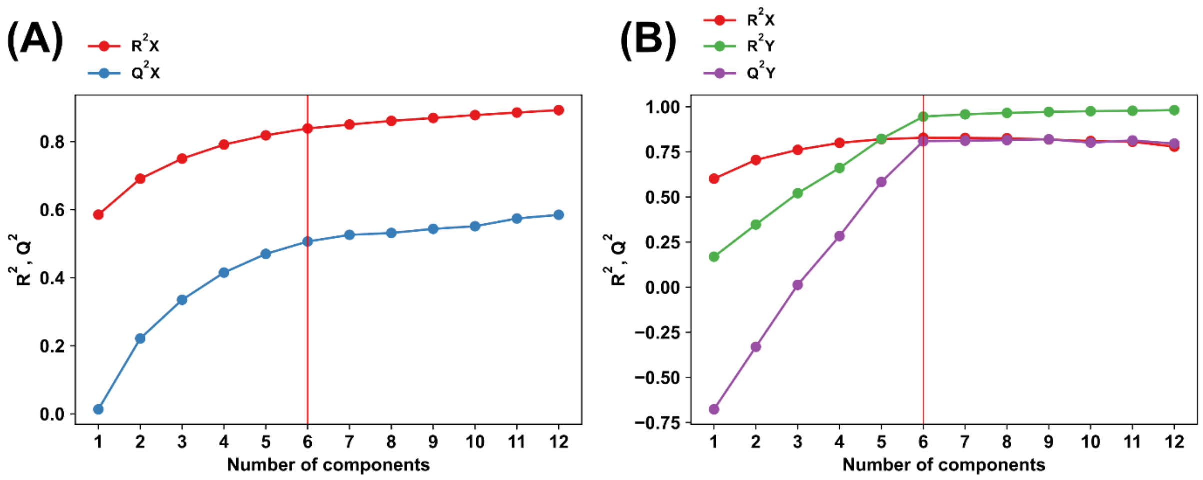

Figure S5: Hyperparameter optimization for endpoint footprinting experiment; scree plots of principial component analysis (A) and PLS-DA (B); Model performance indicator were determined by stratified double 7-fold cross-validation. R^2X describes the goodness of fit or explained variance of the predictor matrix by the model. Q^2X describes the goodness of prediction. R^2Y describes the goodness of fit or explained variance of the response by the model. Q^2Y describes the goodness of prediction for the response. The hyperparameter optimization was stopped, if the increase in Q^2X (A) or Q^2Y (B) was less than 5%

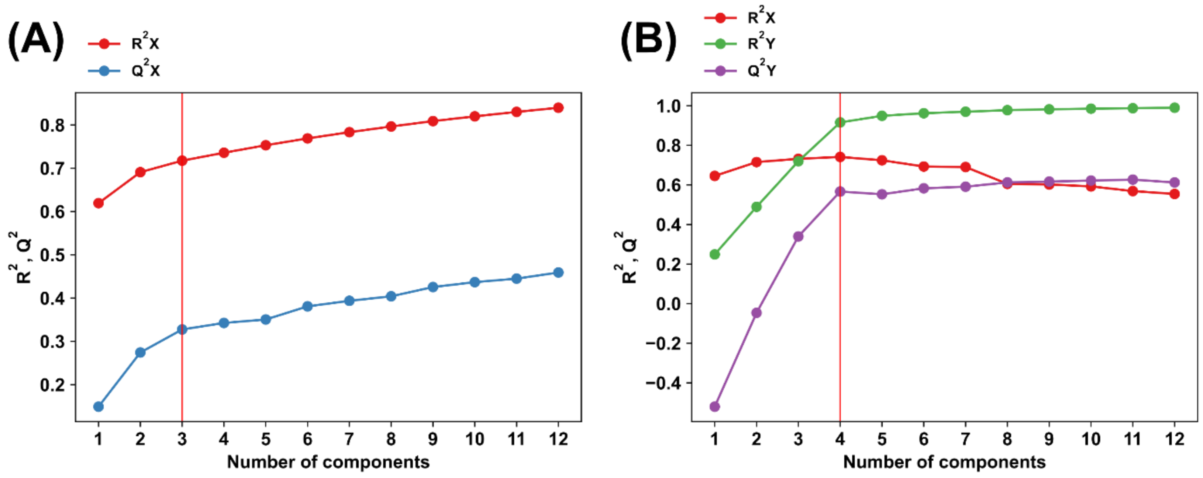

Figure S6: Hyperparameter optimization for time-resolved footprinting experiment; scree plots of principial component analysis (A) and PLS-DA (B); Model performance indicator were determined by stratified double 7-fold cross-validation. R^2X describes the goodness of fit or explained variance of the predictor matrix by the model. Q^2X describes the goodness of prediction. R^2Y describes the goodness of fit or explained variance of the response by the model. Q^2Y describes the goodness of prediction for the response. The hyperparameter optimization was stopped, if the increase in Q^2X (A) or Q^2Y (B) was less than 5%

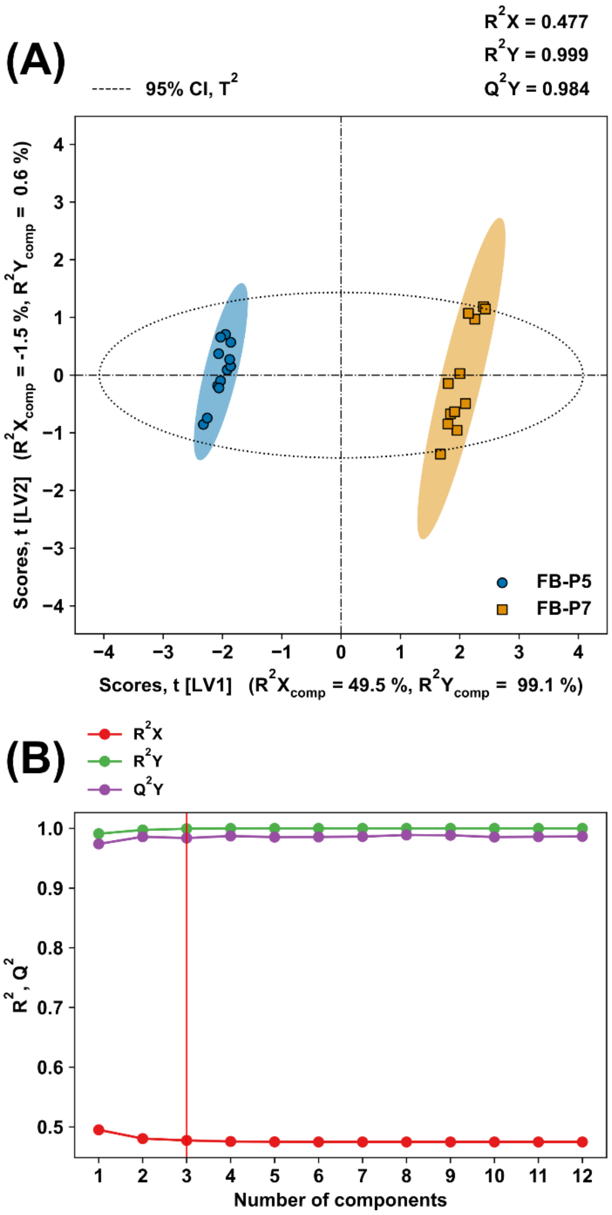

Figure S7: Score plot and scree plot of PLS-DA for PTA; score plot for PLS-DA (A) for P5 and P7; Model performance indicator were determined by stratified double 7-fold cross-validation. R^2X describes the goodness of fit or explained variance of the predictor matrix by the model. Q^2X describes the goodness-of-prediction. R^2Y describes the goodness of fit or explained variance of the response by the model. Q^2Y describes the goodness-of-prediction for the response. (B) Scree plot for hyperparameter optimization; optimization was stopped, if the increase in Q^2Y was less than 5 %

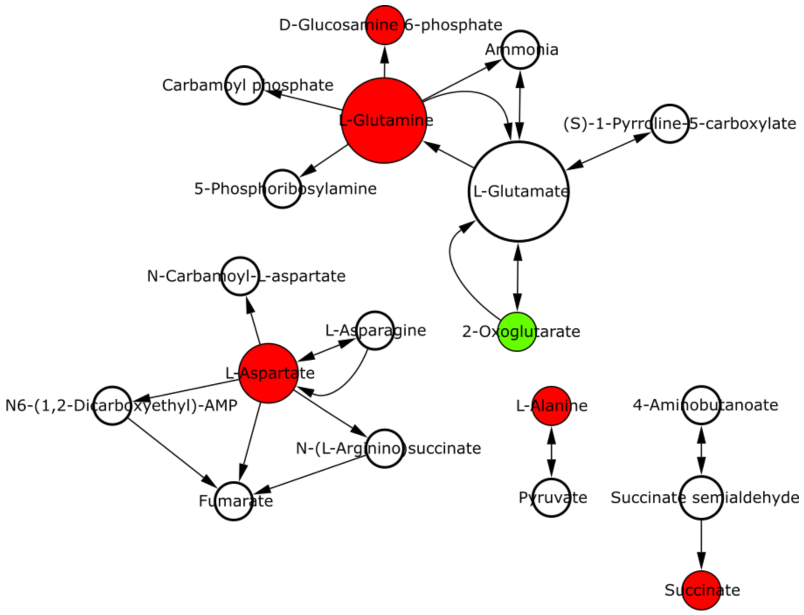

Figure S8: Topology analysis of alanine, aspartate and glutamate metabolism; Directed Graph based on KEGG CGB pathway map. Colored nodes represent significantly changed metabolites between samples FB-P5 and FB-P7. Node color represents higher fold change for FB-P5 (green) or FB-P7 (red) respectively. Node size is relatively scaled based on the normalized betweenness centrality.

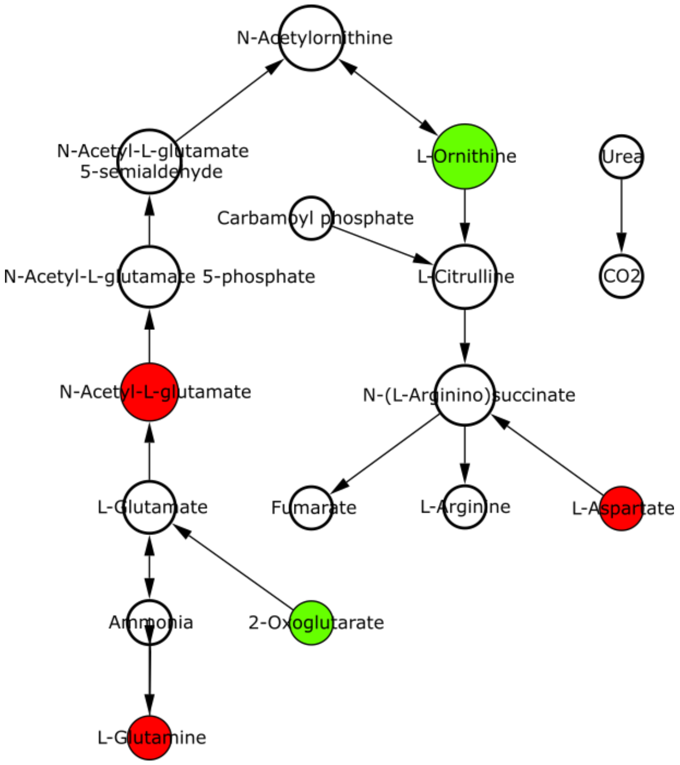

Figure S9: Topology analysis of arginine biosynthesis; Directed Graph based on KEGG CGB pathway map. Colored nodes represent significantly changed metabolites between samples FB-P5 and FB-P7. Node color represents higher fold change for FB-P5 (green) or FB-P7 (red) respectively. Node size is relatively scaled based on the normalized betweenness centrality.

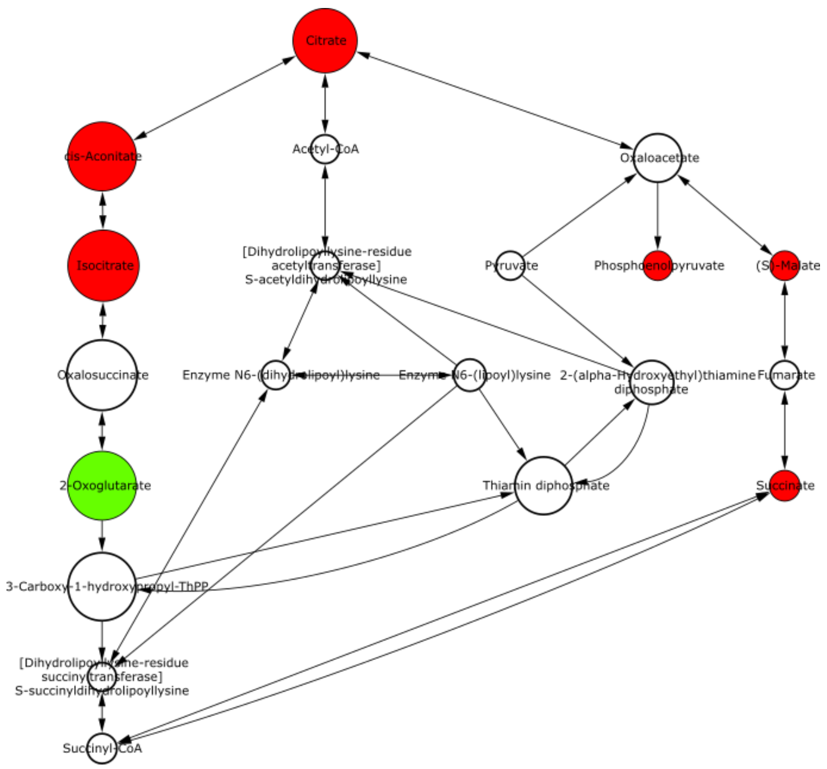

Figure S10: Topology analysis of citrate cycle (TCA cycle); Directed Graph based on KEGG CGB pathway map. Colored nodes represent significantly changed metabolites between samples FB-P5 and FB-P7. Node color represents higher fold change for FB-P5 (green) or FB-P7 (red) respectively. Node size is relatively scaled based on the normalized betweenness centrality.

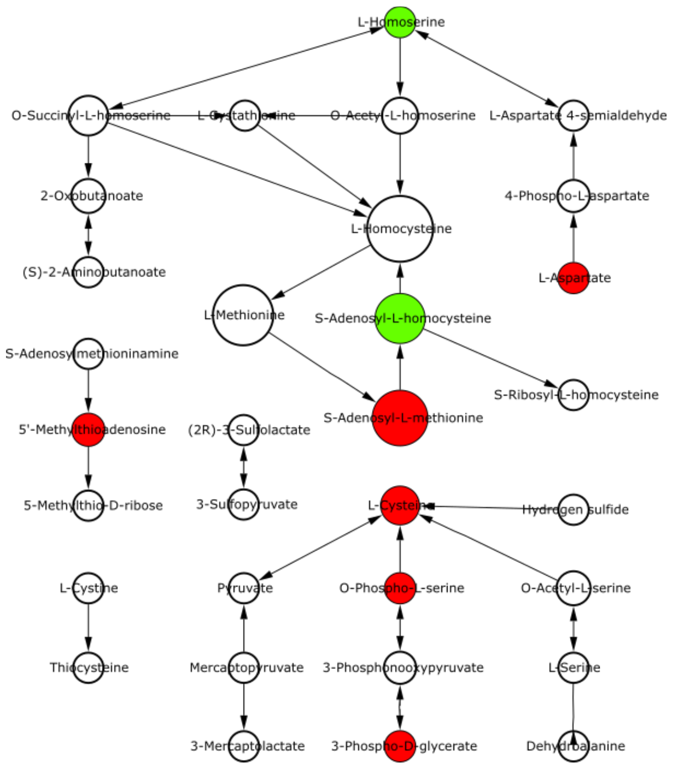

Figure S11: Topology analysis of cysteine and methionine metabolism; Directed Graph based on KEGG CGB pathway map. Colored nodes represent significantly changed metabolites between samples FB-P5 and FB-P7. Node color represents higher fold change for FB-P5 (green) or FB-P7 (red) respectively. Node size is relatively scaled based on the normalized betweenness centrality.

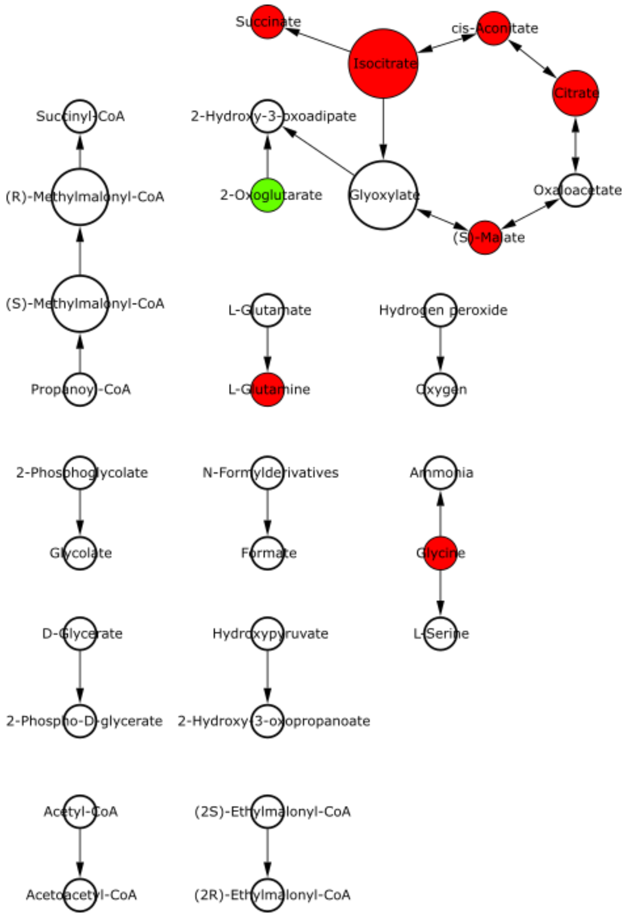

Figure S12: Topology analysis of glyoxylate and dicarboxylate metabolism; Directed Graph based on KEGG CGB pathway map. Colored nodes represent significantly changed metabolites between samples FB-P5 and FB-P7. Node color represents higher fold change for FB-P5 (green) or FB-P7 (red) respectively. Node size is relatively scaled based on the normalized betweenness centrality.

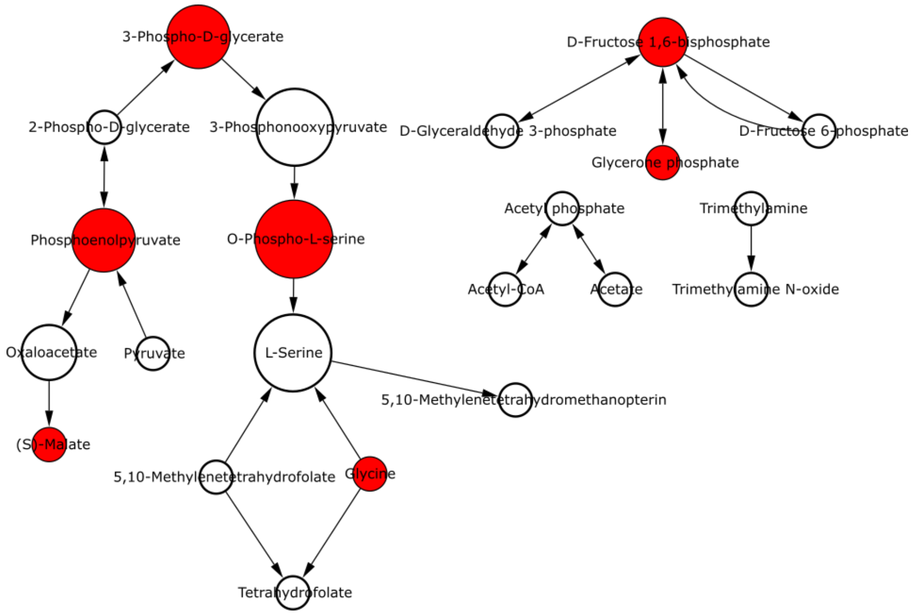

Figure S13: Topology analysis of methane metabolism; Directed Graph based on KEGG CGB pathway map. Colored nodes represent significantly changed metabolites between samples FB-P5 and FB-P7. Node color represents higher fold change for FB-P5 (green) or FB-P7 (red) respectively. Node size is relatively scaled based on the normalized betweenness centrality.

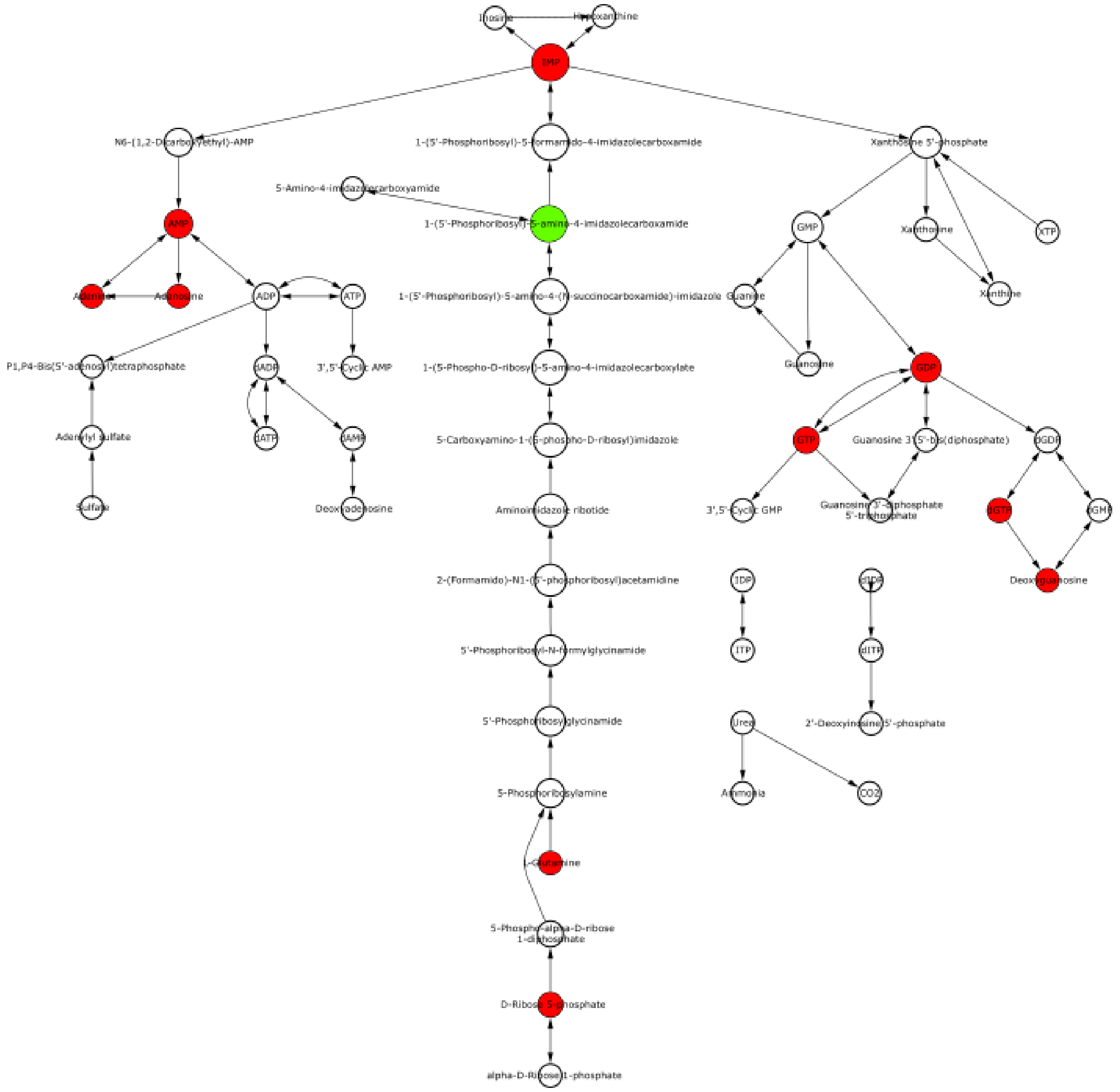

Figure S14: Topology analysis of purine metabolism; Directed Graph based on KEGG CGB pathway map. Colored nodes represent significantly changed metabolites between samples FB-P5 and FB-P7. Node color represents higher fold change for FB-P5 (green) or FB-P7 (red) respectively. Node size is relatively scaled based on the normalized betweenness centrality.

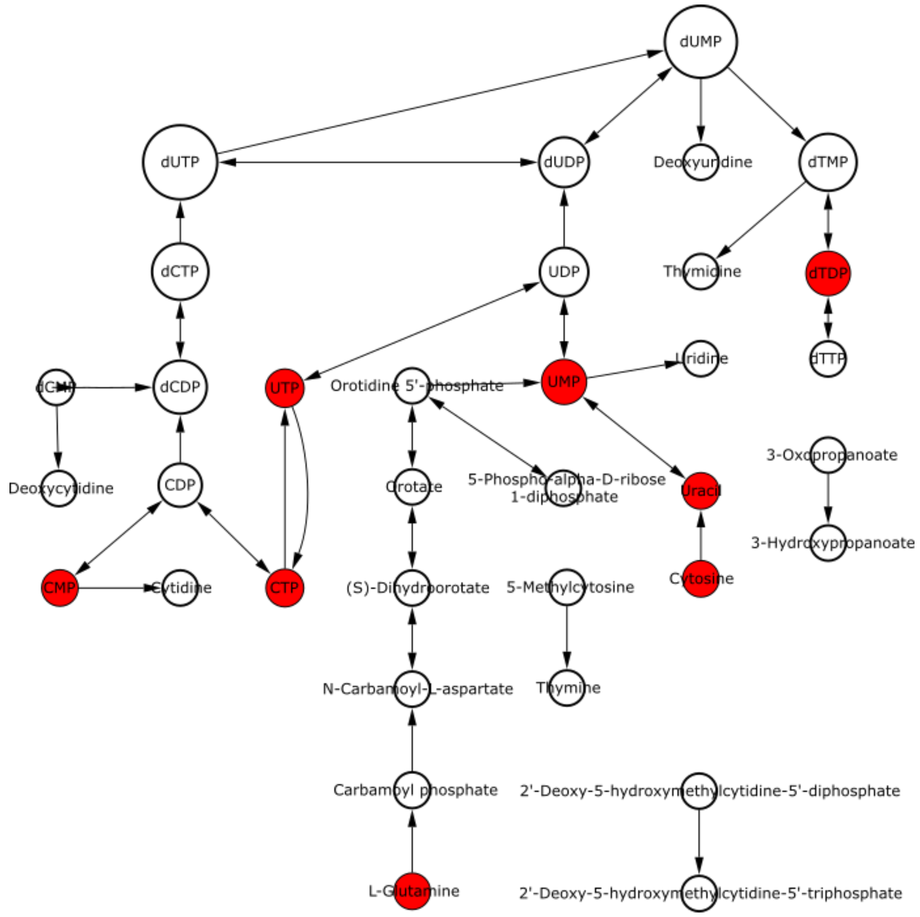

Figure S15: Topology analysis of pyrimidine metabolism; Directed Graph based on KEGG CGB pathway map. Colored nodes represent significantly changed metabolites between samples FB-P5 and FB-P7. Node color represents higher fold change for FB-P5 (green) or FB-P7 (red) respectively. Node size is relatively scaled based on the normalized betweenness centrality.

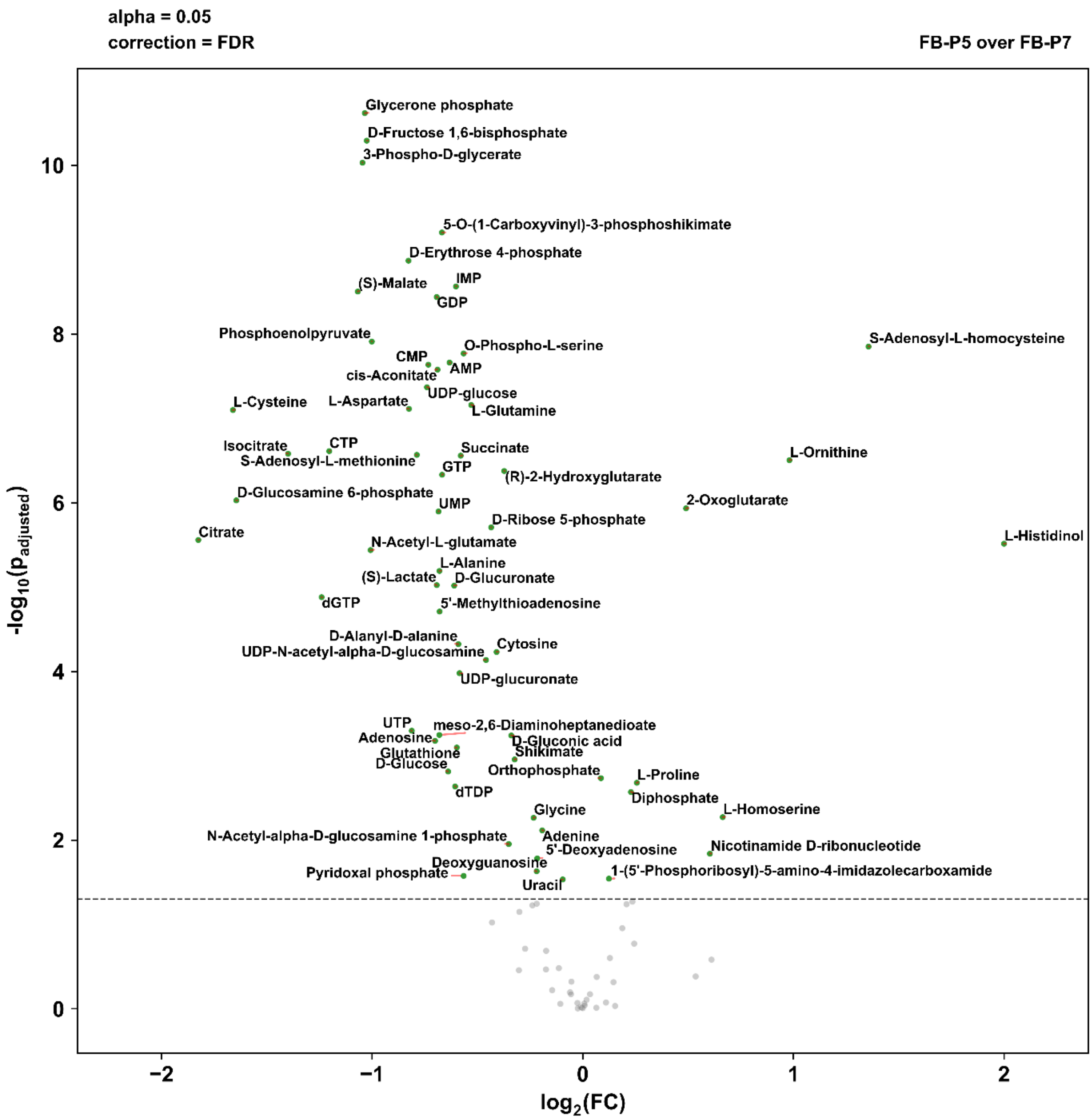

Figure S16: Volcano plot for pathway analysis metabolite list selection. Results of dependent t-test for samples FB-P5 and FB-P7 or with probability of error 0.05 and FDR multi-comparison correction. Fold change > 0 represents higher metabolite abundance in FB-P5

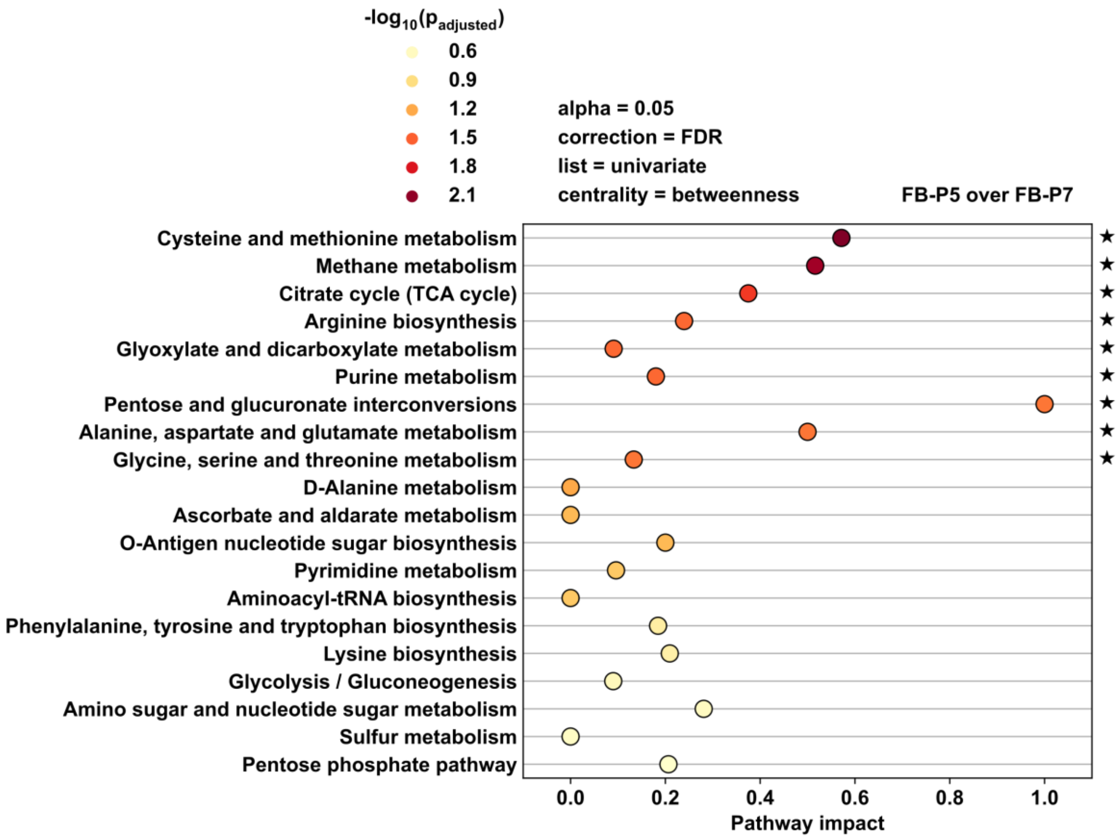

Figure S17: Over-representation analysis and pathway topology analysis validation; for ORA, discriminating metabolites were identified by univariate analysis. Results for the non-limited (FB-P5) and limited cultivation state (FB-P7) and evaluated based on a hypergeometric test for pathway significance with probability of error 0.05 and FDR multi-comparison correction. Significantly changed pathways are indicated with a star. Pathway impact was calculated based on the normalized betweenness centrality measure with respect to KEGG pathway maps. Displayed are 20 of 55 pathways

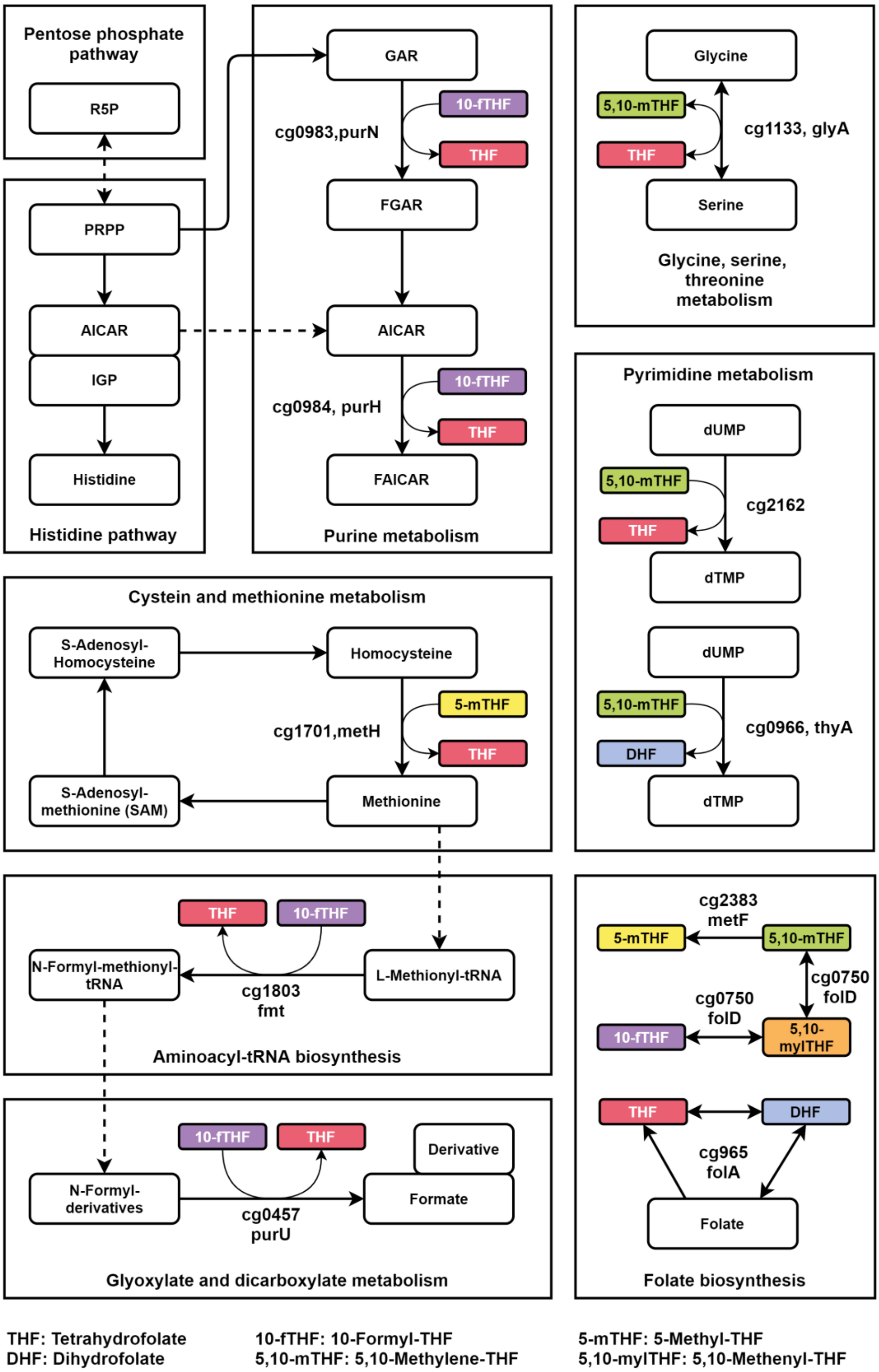

Figure S18: *C. glutamicum* pathways linked to L-histidine biosynthesis; overview of the *C. glutamicum* pathways directly linked to His biosynthesis (pentose phosphate pathway, purine metabolism) or via tetrahydrofolate cofactor regeneration (Glycine, serine and threonine metabolism, pyrimidine metabolism, cystein and methionine metabolism, aminoacyl-tRNA biosynthesis, glyoxylate and dicarboxylate metabolism, folate biosynthesis) based on KEGG pathway maps (CGB). THF derivatives are colored consistently across the pathways
