# Supplementary figures and images for: Rapid exometabolome footprinting combined with multivariate statistics: A powerful tool for bioprocess optimization
Source: Eng Life Sci. 2024 Mar 5;25(2):2300222. doi: 10.1002/elsc.202300222 (PMC11842285; doi:10.1002/elsc.202300222)

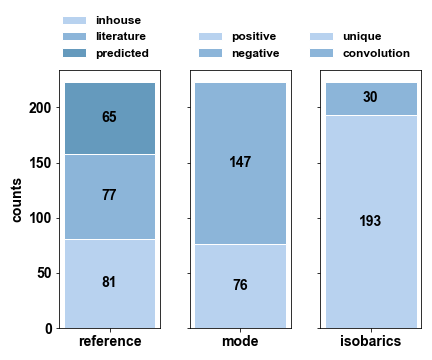

Supplement: Supplementary file 2 — Supporting Information [file ELSC-25-2300222-s002.zip › Supplement-to-Reiter-et-al.-2023a-main/examples/Database/Development/Projects/cgb/cgb/summary_development.png]
